# Heterotrophic Bacterioplankton Growth and Physiological Properties in Red Sea Tropical Shallow Ecosystems With Different Dissolved Organic Matter Sources

**DOI:** 10.3389/fmicb.2021.784325

**Published:** 2022-01-03

**Authors:** Luis Silva, Maria Ll. Calleja, Tamara M. Huete-Stauffer, Snjezana Ivetic, Mohd I. Ansari, Miguel Viegas, Xosé Anxelu G. Morán

**Affiliations:** ^1^Division of Biological and Environmental Sciences and Engineering, Red Sea Research Center, King Abdullah University of Science and Technology, Thuwal, Saudi Arabia; ^2^Department of Climate Geochemistry, Max Planck Institute for Chemistry, Mainz, Germany; ^3^Department of Biosciences, Integral University, Lucknow, India; ^4^Centro Oceanográfico de Gijón/Xixón (IEO, CSIC), Gijón, Spain

**Keywords:** seagrass, mangrove, phytoplankton, heterotrophic bacteria, growth rates, dissolved organic matter, coastal lagoon, Red Sea

## Abstract

Despite the key role of heterotrophic bacterioplankton in the biogeochemistry of tropical coastal waters, their dynamics have been poorly investigated in relation to the different dissolved organic matter (DOM) pools usually available. In this study we conducted four seasonal incubations of unfiltered and predator-free seawater (Community and Filtered treatment, respectively) at three Red Sea coastal sites characterized by different dominant DOM sources: Seagrass, Mangrove, and Phytoplankton. Bacterial abundance, growth and physiological status were assessed by flow cytometry and community composition by 16S rRNA gene amplicons. The Seagrass site showed the highest initial abundances (6.93 ± 0.30 × 10^5^ cells mL^–1^), coincident with maximum DOC concentrations (>100 μmol C L^–1^), while growth rates peaked at the Mangrove site (1.11 ± 0.09 d^–1^) and were consistently higher in the Filtered treatment. The ratio between the Filtered and Community maximum bacterial abundance (a proxy for top-down control by protistan grazers) showed minimum values at the Seagrass site (1.05 ± 0.05) and maximum at the Phytoplankton site (1.24 ± 0.30), suggesting protistan grazing was higher in open waters, especially in the first half of the year. Since the Mangrove and Seagrass sites shared a similar bacterial diversity, the unexpected lack of bacterial response to predators removal at the latter site should be explained by differences in DOM characteristics. Nitrogen-rich DOM and fluorescent protein-like components were significantly associated with enhanced specific growth rates along the inshore-offshore gradient. Our study confirms the hypotheses that top–down factors control bacterial standing stocks while specific growth rates are bottom-up controlled in representative Red Sea shallow, oligotrophic ecosystems.

## Introduction

In the tropical, oligotrophic oceans the role of small autotrophic (mostly cyanobacteria) and heterotrophic (bacteria and archaea) prokaryotes is fundamental for carbon budgets and fluxes (Bock et al., 2018; Richardson, 2019). In particular, heterotrophic prokaryotes [hereafter heterotrophic bacteria since archaea represent a minor contribution in surface waters (Karner et al., 2001)] are the main recycling organisms of dissolved organic matter (DOM). Through their uptake of DOM, ultimately derived from primary producers and their subsequent consumption by protistan grazers, bacteria are an important link for higher organisms in marine food webs, with consequences for biogeochemical cycling (Azam et al., 1983; Azam and Malfatti, 2007; Pomeroy et al., 2007; Kirchman et al., 2009). In shallow tropical waters, DOM can be autochthonous [i.e., produced within the system by phytoplankton (Nagata, 2000; Lønborg et al., 2009a) and macrophytes such as seagrasses, mangroves or macroalgae (Bertilsson and Jones, 2003; Cawley et al., 2012)], or allochthonous (i.e., transported from elsewhere, either nearby marine areas, groundwater, rivers or atmospheric deposition; Sobczak et al., 2005; Cawley et al., 2012; Rushdi et al., 2019). DOM can be easily transported, and autochthonous sources can ultimately work as allochthonous sources in nearby regions (Alongi and Mukhopadhyay, 2015). In this regard, changes in the relative contribution of the different autochthonous (phytoplankton or macrophytes) sources of DOM (Stepanauskas et al., 2000; Säwström et al., 2016) can affect the carbon flux in tropical marine food webs, especially through microbes (Lønborg et al., 2018).

Resource availability (bottom–up control) and mortality by protistan grazers and viruses (top–down control), together with other environmental drivers among which temperature stands out, are the main factors constraining bacterial abundance and metabolism in aquatic ecosystems everywhere (e.g., White et al., 1991; Ducklow and Carlson, 1992; Jürgens and Massana, 2008; Morán et al., 2010; Vaqué et al., 2014). However, few investigations have taken place in low latitude environments, either coastal or oceanic. The reduced response of heterotrophic prokaryotes production rates to warm temperatures in offshore subtropical and tropical waters was recently associated with either strong resource limitation or intense protistan grazing and/or viral lysis (Morán et al., 2017). Many gaps though remain in our understanding of the factors constraining microbial plankton stocks and activity in tropical coastal regions, especially those affected by the key low latitude macrophyte ecosystems: mangroves and seagrasses. While phytoplankton are the dominant primary producers in pelagic ecosystems worldwide, their typically oligotrophic nature in tropical waters may be substantially alleviated by DOM exported from mangrove forests and seagrass meadows. Furthermore, due to their extensive geographical coverage, both types of vegetated coastal ecosystems represent an important contribution to global carbon fluxes worldwide (Kristensen et al., 2008; Alongi and Mukhopadhyay, 2015).

The Red Sea is one of those poorly studied oligotrophic regions. Its central part is generally characterized by low pelagic primary productivity (Raitsos et al., 2013), mostly caused by a quasi-permanent pycnocline preventing the influx of deep, nutrient-rich water into the euphotic zone, but also by the low fertilization from terrestrial runoff, virtually non-existent or episodic after the rare rainstorms (Manasrah et al., 2019). In fact, in the absence of permanent rivers, the allochthonous inputs of nutrients in the central Red Sea is restricted to dust events (Sheppard et al., 1992), urban areas sewage, and material derived from coastal macrophytes (Alongi and Mukhopadhyay, 2015). Comparable to other tropical areas, pico- and nanoplankton contribute up to 80% of Red Sea pelagic primary production (Brewin et al., 2019; Qurban et al., 2019), therefore representing the main source of DOM in macrophyte-free waters. Clarifying the manner in which DOM and heterotrophic bacteria interact along the Red Sea coastline is thus vital for a better understanding the fate of the different autochthonous sources (Nagata, 2000). The balance between bacterial biomass production (BP) and total carbon demand (BCD, used for production and respiration) is represented by the variable bacterial growth efficiency (BGE = BP/BCD), a useful means of assessing the role of heterotrophic bacterioplankton in aquatic ecosystems (del Giorgio and Cole, 1998; Carlson et al., 2007). Most available studies on DOM transformations by heterotrophic bacteria are strongly leaned to production rather than respiration and therefore BGE estimates in tropical regions are scarce (Lønborg et al., 2019).

In order to improve our knowledge about how different autochthonous DOM sources affect bacterioplankton metabolism in the central Red Sea, we conducted four short-term (4–6 days) incubations of epipelagic samples from three sites, each dominated by one of the major DOM sources found in tropical regions (seagrasses, mangroves, and phytoplankton), covering a full seasonal cycle, from February 2016 to December 2017. Incubations were performed with and without the presence of protistan grazers and other organisms larger than 1.2 μm in order to elucidate the relative importance of bottom–up and top–down controls on bacterioplankton standing stocks and the growth rates of different bacterial physiological groups (del Giorgio and Gasol, 2008). As possible factors constraining bacterial responses, we included temperature and inorganic nutrients, DOM (bulk DOC and DON and fluorescent components) and phytoplankton (chlorophyll *a*) concentrations. The prokaryotic community composition at the three sites was determined through the analysis of 16S rRNA genes. A research on the combined effects of mangrove- and seagrass-originated DOM and temperature in the Great Barrier Reef reported higher exoenzymatic activity, carbon fluxes and growth rates of bacteria exposed to those substrates, especially to mangrove-originated DOM (Baltar et al., 2017; Lønborg et al., 2019; Morán et al., 2020). Based on these and other recent studies conducted in nearby shallow Red Sea waters (Silva et al., 2019; Sabbagh et al., 2020), our hypotheses in this study were that: (i) exposition to seagrass- and mangrove-originated DOM would result in higher bacterial growth rates compared with that released by phytoplankton; (ii) protistan grazing would emerge as the main control of heterotrophic bacterial abundances *in situ*, and (iii) phytoplankton-originated DOM would yield the lowest BGE values.

## Materials and Methods

### Study Sites and Experimental Design

**FIGURE 1:**
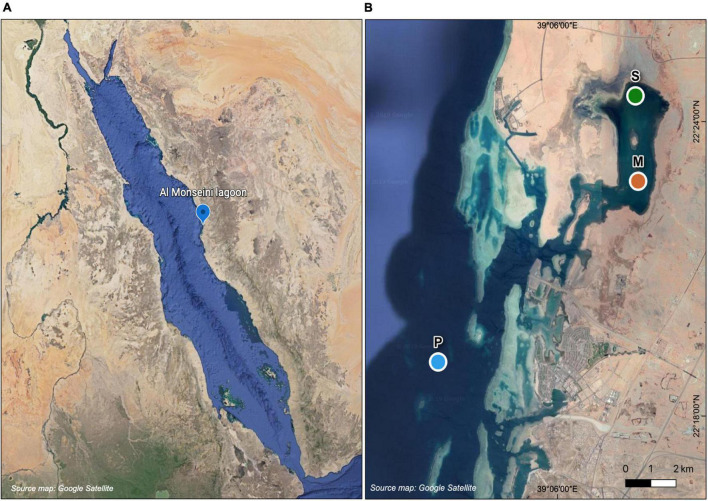
Map of the Red Sea showing the study site **(A)** and detailed location of the sampling stations at Al Monseini coastal lagoon and nearby coastal waters **(B)**: Seagrass-dominated site (S), Mangrove-dominated site (M), and Phytoplankton-dominated site (P).

Surface (ca. 0.5 m) water was collected from three different sites along an inshore-offshore gradient in a coastal lagoon (Al Monseini), situated between King Abdullah University of Science and Technology (KAUST) and King Abdullah Economic City (KAEC), in the eastern coast of the central Red Sea (Figure 1). The three sites were dominated by different primary producers, two of them benthic macrophytes (one overlying *Cymodocea serrulata* seagrass meadows and the other in the vicinity of a patch of *Avicennia marina* mangroves) and the other one pelagic (phytoplankton). Hereinafter we will use the names Seagrass, Mangrove and Phytoplankton or their initials (S, M, and P) when referring to each site. The Seagrass (22°24.5′ N, 39°7.8′ E) and Mangrove (22°22.8′ N; 39°7.9′ E) sites were located inside the coastal lagoon, with depths of 1.5–2 m, and the Phytoplankton (22°19.1′ N, 39°3.2′ E, ca. 20 m) site was located offshore the lagoon entrance (Figure 1). Aiming at covering the seasonal cycle, sampling dates were staggered every 2–3 months. Actual samplings took place on 15 February 2016, 1 June 2016, 5 September 2016, and 4 December 2017. Temperature and salinity were measured immediately prior to sampling using a CTD (conductivity, temperature and depth) unit (Valeport MONITOR CTD + 500) and water sampling was performed by submerging two acid-washed polycarbonate carboys of 9 L per site.

For each site, experimental incubations of 4–6 days were performed following the seawater culture method (Ammerman et al., 1984). Two different treatments were used for monitoring bacterial and DOM dynamics at each site: unfiltered water was incubated in order to assess the dynamics of the entire microbial community (Community treatment) and water pre-filtered through pre-combusted Whatman GF/C filters (1.2 μm nominal size) was incubated to target the direct interaction between heterotrophic bacteria and DOM after removing protistan grazers and phytoplankton, virtually retained completely onto the filters (Filtered treatment). Incubations were performed in triplicates in 2 L acid-washed polycarbonate bottles in temperature-controlled incubators (Percival – I-22LLVL) with *in situ* light (12 h light:12 h dark cycle was set for all incubations, as in that Saudi Arabia location there are minor changes between light hours year-round) and *in situ* temperature conditions (which were adjusted for each site and season). The aim of the diel cycle of light and darkness in the Filtered treatment was to allow for photoheterotrophy of the natural bacterioplankton assemblages. Samples for inorganic nutrients, dissolved organic carbon (DOC) and nitrogen (DON) concentrations, DOM fluorescence (FDOM) components, bacterial abundance and bacterial physiological properties were collected twice per day to better capture the exponential phase of growth until the abundance of bacteria reached a plateau or started to drop (typically after 1 or 2 days). Incubations lasted up to 6 days, and were stopped when a stationary or decay phase was well-established. We focused on the initial responses and did not consider any secondary growth phases in our calculations, which were inconsistently detected.

Ambient size-fractionated chlorophyll *a* (Chl *a*) concentration was measured at each sampling site after sequential filtration of 200 ml samples through polycarbonate (Isopore™ membrane filters) filters of 20, 2, and 0.2 μm pore-size in order to respectively retrieve the micro-, nano-, and picoplankton size-fractions. Filters were kept at –80°C until analysis. Chl *a* was extracted in 90% acetone for 24 h in dark conditions at 4°C. A Turner Trilogy fluorometer was used to measure Chl *a*, using the acidification method (Holm-Hansen and Riemann, 1978) calibrated with a chlorophyll *a* standard (from *Anacystis nidulans*, Sigma Aldrich). Total Chl *a* was calculated by summing the three size-fractions.

### Inorganic Nutrients and Dissolved Organic Matter

Samples for nutrient analyses were collected in 15 mL sterile polypropilene Falcon tubes after being filtered through 0.2 μm Millipore^®^ polycarbonate filters and stored frozen at –20°C until analysis. A segmented flow analyzer from Seal Analytical^®^ was used to analyze nitrate (NO_3_^–^), nitrite (NO_2_^–^), and phosphate (PO_4_^3–^). Detection limits were 0.2, 0.06, and 0.01 μmol L^–1^ for NO_3_^–^, NO_2_^–^, and PO_4_^3–^, respectively. Standards were prepared with a nutrient-free artificial seawater matrix in acid-washed glassware. Consumption and production rates of inorganic nutrients (μmol L^–1^ d^–1^) were estimated as the slope of the linear model of nutrient concentrations vs. time during the bacterioplankton exponential growth phases. Positive and negative values correspond to production and consumption, respectively.

Samples for DOC and total dissolved nitrogen (TDN) were filtered through 0.2 μm Millipore^®^ polycarbonate filters. To avoid contamination, filters were pre-washed with 50 mL of 1.2 mol L^–1^ hydrochloric acid and rinsed with abundant MQ before sample filtration. The initial filtrate was discarded and the subsequent filtrate was used for sample storage and analysis. Each filtered sample was acidified with H_3_PO_4_ and kept at 4°C until further analysis by high temperature catalytic oxidation (HTCO) using a Shimadzu TOC-L. Reference materials of deep-sea carbon (42–45 μmol C L^–1^ and 31–33 μmol N L^–1^) and low carbon water (1–2 μmol C L^–1^) were used to monitor the accuracy of DOC and TDN concentration measurements. DON concentrations were calculated after subtracting the dissolved inorganic nitrogen (DIN) to the TDN (DON = TDN – DIN), where DIN (μmol C L^–1^) = [NO_3_^–^] + [NO_2_^–^].

### Dissolved Organic Matter Fluorescence Measurements

Samples for DOM fluorescence analyses were filtered through 0.2 μm Millipore^®^ polycarbonate filters and measured immediately after filtration. UV-VIS fluorescence spectroscopy was measured using a HORIBA Jobin Yvon AquaLog spectrofluorometer with a 1 cm path length quartz cuvette. Three-dimensional fluorescence excitation emission matrices (EEMs) were recorded by scanning with an excitation range between 240 and 600 nm and an emission wavelength range of 250–600 nm, both at 3 nm increments and using an integration time of 8 s. To correct and calibrate the fluorescence spectra post-processing steps were followed according to Murphy et al. (2010), in which Raman-normalized Milli-Q blanks were subtracted to remove the Raman scattering signal (Stedmon et al., 2003). All fluorescence spectra were Raman area (RA) normalized by the subtraction of daily blanks performed using Ultra-Pure Milli-Q sealed water (Certified Reference, Starna Cells). Inner-filter correction (IFC) was also applied according to McKnight et al. (2001). MATLAB (version R2015b) was used for the RA normalization, blank subtraction, IFC and generation of EEMs. The EEMs obtained were subjected to PARAFAC modeling using drEEM Toolbox (Murphy et al., 2013). Before the analysis, Rayleigh scatter bands [first order at each wavelength pair where Ex = Em ± bandwidth; second order at each wavelength pair where Em = 2 Ex ± (2 × bandwidth)] were trimmed. Only one sample was identified as an outlier and the model was validated using half split validation and random initialization (Stedmon and Bro, 2008). The data array consisted of 568 samples that were split into two random halves of 284 EEMs each. The nested PARAFAC algorithm was then applied stepwise to both data arrays for 2–7 components. Ten iterations and a convergence criterion of 1e-6 were used for each model. The spectral properties of the components derived from each half were compared and found to be congruent according to the Tucker Coefficient described in Lorenzo-Seva and Ten Berge (2006) for a four-component model. The maximum fluorescence (Fmax) is reported in Raman units (RU) (Stedmon et al., 2003; Murphy et al., 2010). The four components from the validated model are: peak C1 at Ex_1_(Ex_2_)/Em 252(324)/397 nm (humic-like peak M, Coble, 2007), peak C2 at Ex_1_(Ex_2_)/Em 252(366)/467 nm (marine humic-like peak C, Coble, 2007), peak C3 at Ex/Em 303/337 [protein-like peak T (Coble, 2007) attributed to Tryptophan], and peak C4 at Ex/Em 270/312 nm [corresponds to protein-like peak B (Coble, 2007) and attributed to Tyrosine]. Consumption and production rates of each DOM fluorescent components (ΔRU/Δt, RU d^–1^) were estimated as the slope of the fluorescence intensity vs. time during the exponential growth phases. Positive and negative values were considered as production and consumption, respectively. The percentage of protein-like compounds was calculated for each EEM as the sum of the fluorescence intensity of peaks C3 and C4 divided by the sum of fluorescence intensity of all peaks: (Fmax _C3_ + Fmax _C4_)/(Fmax _C1_ + Fmax _C2_ + Fmax _C3_ + Fmax _C4_) × 100.

### Single-Cell Physiological Groups of Heterotrophic Bacteria

We followed the methodology described in more detail in Gasol and Morán (2015) to characterize the physiological structure of heterotrophic bacteria at the three sites by considering five different single-cell groups (del Giorgio and Gasol, 2008): cells with low and high nucleic acid content (LNA and HNA, respectively), cells with healthy and damaged membranes (*Live* and *Dead*, respectively) and actively respiring cells (CTC+). LNA and HNA cells were differentiated according to relative nucleic acid content based on their green fluorescence signal after being stained with SybrGreen (Marie et al., 1997). Samples to distinguish these two groups were previously fixed with 1% paraformaldehyde + 0.5% glutaraldehyde (final concentration), deep-frozen in liquid nitrogen and stored at –80°C until analysis. Samples were thawed, stained and run in a flow cytometer within 1–3 months after collection. Membrane-intact (*Live*) and membrane-compromised (*Dead*) cells were assessed by combining two nucleic acid stains, SybrGreen (Molecular Probes) and propidium iodide (PI, Sigma Chemical Co), yielding green and red fluorescence signals, respectively. PI can only penetrate into cells with large holes in their membranes. *Live* and *Dead* cells were analyzed *in vivo* as described in Grégori et al. (2001). Actively respiring cells (CTC+), able to reduce the tetrazolium salt 5-cyano-2,3-ditolyl tetrazolium chloride or CTC, were distinguished by the red fluorescence signal resulting from the deposition of oxidized crystals of CTC (Sherr et al., 1999). CTC+ cells are *Live* cells representing typically a subset of the HNA group. This group was analyzed *in vivo* after 90 min incubation in the dark with the compound. All samples were analyzed in a BD FACSCanto II flow cytometer.

### Bacterial Abundance and Biomass

The abundances of the five physiological groups were calculated after gravimetric calibration of the flow cytometer flow rates. Total bacterial abundances correspond to the sum of LNA and HNA cells described in the previous section. Filtration by GF/C filters resulted in an overall mean loss (i.e., retention onto the filters) of 26 ± 10% (SD) of the heterotrophic cells present in the Community treatment. From a seasonal perspective, retention was minimum in September (14 ± 9%) and maximum in February (32 ± 5%). Latex fluorescent beads of 1 μm diameter (Molecular Probes, ref. F-13081) were added to each sample as an internal standard. Side scatter (SSC, light scatter at 90°) values relative to the fluorescent beads were converted into cell diameter following the empirical calibration of Calvo-Díaz and Morán (2006). Cell diameter was then converted to biovolume (Bv) and finally converted into bacterial biomass (BB) using the formula of Gundersen et al. (2002): fg C cell^–1^ = 108.8 × [Bv]^0^.^898^ and then converted into μmol C L^–1^.

### Growth Rates and Carrying Capacities

The growth rates (μ) of four physiological groups (LNA, HNA, *Live* and CTC+ cells) were calculated as the slope of the natural logarithm of bacterial abundances vs. time during the corresponding exponential growth phases. Total heterotrophic bacterial growth rates correspond to the changes in abundance of total bacteria [LNA + HNA (reversed order for consistency)]. Viruses were unlikely to affect bacterial growth rates due to the short-time of the exponential growth periods (1–2 days, Guixa-Boixareu et al., 1999). Therefore, we will use the terms “specific growth rate” for the Filtered treatment and “net growth rate” for the Community treatment, containing the entire microbial food web (i.e., with protistan grazers). Carrying capacity is defined here as the maximum abundance recorded for each bacterial group at the plateau stage of the incubations. The ratio between the Filtered and the Community treatment carrying capacities was used as a proxy of top–down control.

### Bacterial Growth Efficiency

Bacterial growth efficiencies (BGE, %) were estimated by following changes in DOC concentrations (ΔDOC/Δt) and bacterial biomass (ΔBB/Δt), during the exponential growth phase, as shown in the following formula: BGE (%) = [(ΔBB/Δt)/(ΔDOC/Δt)] × 100, where ΔBB/Δt represents the increase of total bacterial biomass (μmol C L^–1^ d^–1^), and ΔDOC/Δt is the carbon consumption (μmol C L^–1^ d^–1^) estimated from the decrease in total DOC.

### Prokaryotic Diversity

Five liter of each site were filtered through 0.2 μm Sterivex filters for each sampling period (12 samples in total), frozen and extracted with the Power Soil DNAeasy kit (Qiagen). The regions V4-V5 of the 16S rRNA gene were amplified using the primers 515F-Y and 926R (Parada et al., 2016). Sequencing of 2 × 300 bp was performed on a MiSeq platform, where all steps performed and reagents used followed the MiSeq Illumina protocol. After trimming of indexes with cutadapat 2.3 (Martin, 2011), the clean sequences were analyzed with the DADA2 pipeline (Callahan et al., 2016, 2017) and the taxonomy was assigned from the reference database SILVA 132 at 80% bootstrapping. In total, 5950 final amplicon sequence variants (ASVs) were produced after removal of chimeras, singletons, and sequences assigned to mitochondria, chloroplasts or wrongly assigned to phyla different than Archaea or Bacteria. Samples were normalized to the minimum number of reads (21423) for all subsequent analyses.

### Data Analysis

JMP PRO 14 and R (version 3.6.1) software were used for statistical analyses. Repeated measurements analyses of variance (ANOVA), considering the month nested to station and *post hoc* comparisons between levels were performed using R “psycho” package (Makowski, 2018). Two-way ANOVAs were used to check for differences between bacterial physiological growth rates. Pearson *r* coefficients are given for correlation analyses. Sequence processing was performed with the dada2 package (Callahan et al., 2016) while diversity estimates and multidimensional scaling were calculated with vegan (Oksanen et al., 2019).

## Results

### Environmental Conditions and Resource Availability

**FIGURE 2:**
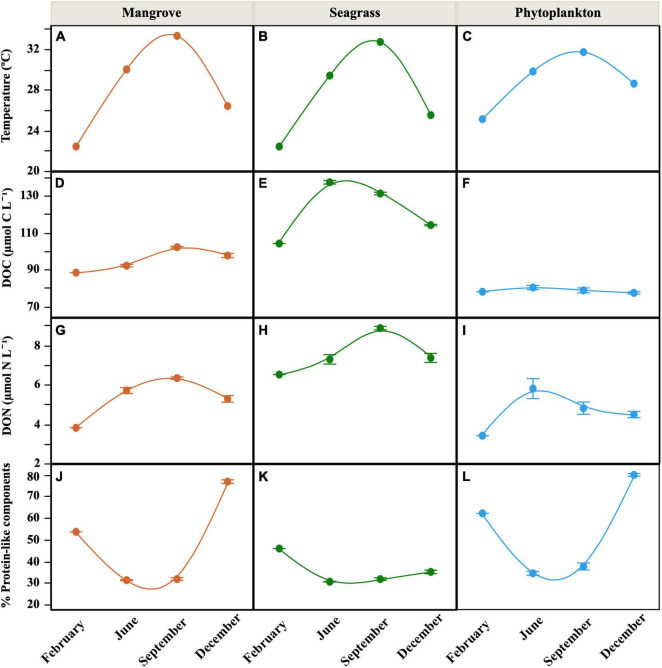
Seasonal distribution of mean (± SE) *in situ* values of temperature **(A–C)**, DOC concentration **(D–F)**, DON concentration **(G–I),** and percent contribution of protein-like components to total FDOM **(J–L)** at the three sites. Smooth fitting joins site points for clarity.

Warm temperatures were found year-round in the lagoon (Seagrass and Mangrove) and offshore water (Phytoplankton) sites, though showing a clear seasonality from 22.4°C in February to 33.3°C in September (Figures 2A–C). Salinity at the Phytoplankton site was relatively stable (39.3–39.5) while both the Seagrass and Mangrove sites showed higher variability (37.1–41.1 and 38.5–41.0, respectively). Total chlorophyll *a* concentrations were low to moderate (0.10–1.35 μg L^–1^), with the maximum observed at the Seagrass site in June and the lowest values systematically detected at the Phytoplankton site (Table 1). The contribution of picophytoplankton (0.2–2 μm) ranged from 4 to 79%, while that of nanophytoplankton (2–20 μm) ranged from 10 to 62% (data not shown). Microphytoplankton (>20 μm) contribution to total chlorophyll *a* was the lowest of the three size-fractions in 58% of the samples (with a minimum value of 3.0%), but it made up to 74 and 87% inside the lagoon (Mangrove and Seagrass sites, respectively) in June.

**TABLE 1:** Mean (± SE) concentrations of total chlorophyll *a*, inorganic nutrients and the C:N ratio of DOM at the onset of the incubations of water samples from the three sites.

	Chl *a* (μg L^–1^)			Nitrite (μmol L^–1^)			Nitrate (μmol L^–1^)			Phosphate (μmol L^–1^)			DOM C:N ratio		
	S	M	P	S	M	P	S	M	P	S	M	P	S	M	P
February	0.26	0.33	0.14	0.286	0.043	0.054	0.12	0.29	0.32	n.d.	n.d.	n.d.	16.0	23.2	22.9
June	1.35	0.53	0.14	0.023 ± 0.007	0.037 ± 0.004	0.028 ± 0.002	0.03 ± 0.01	0.21 ± 0.03	0.18 ± 0.03	0.010 ± 0.004	0.018 ± 0.001	0.018 ± 0.000	18.9 ± 0.7	16.2 ± 0.5	14.0 ± 1.2
September	0.41	0.41	0.08	0.041 ± 0.011	0.079 ± 0.006	0.060 ± 0.038	0.12 ± 0.01	0.84 ± 0.01	0.14 ± 0.04	0.039 ± 0.012	0.032 ± 0.006	0.050 ± 0.022	14.8 ± 0.1	16.1 ± 0.2	16.6 ± 1.3
December	0.38	0.35	0.26	0.066 ± 0.008	0.076 ± 0.007	0.013 ± 0.000	0.64 ± 0.04	1.04 ± 0.06	0.38 ± 0.07	0.039 ± 0.015	0.063 ± 0.014	0.052 ± 0.020	15.6 ± 0.5	18.6 ± 0.7	17.3 ± 0.7

*S, Seagrass; M, Mangrove; P, Phytoplankton. n.d., no data.*

The minimum and maximum inorganic nutrient concentrations at the onset of the incubations were similar for both nitrate and phosphate, with minima in June at Seagrass and maxima in December at Mangrove (Table 1). Contrary to inorganic nutrients, the Seagrass site showed much higher DOM concentrations than the other sites. DOC concentration averaged 121.6 ± 4.0 μmol C L^–1^ there, with the Phytoplankton site consistently characterized by the lowest values (repeated measurements ANOVA, *p* < 0.001, *n* = 30). Overall, DOC concentration ranged between 77.2 and 137.3 μmol C L^–1^ (Figures 2D–F) and it was positively correlated with temperature inside the lagoon (*r* = 0.89, *p* < 0.0001 at the Seagrass site; *r* = 0.74, *p* = 0.006 at the Mangrove site, *n* = 10). The concentration of DON (3.4–8.9 μmol N L^–1^, Figures 2G–I) followed a similar pattern, with the Seagrass site showing the highest values and the Phytoplankton site the lowest. The corresponding C:N ratios ranged from 14 to 23 without any clear geographical difference (Table 1). Positive correlations between DON concentration and temperature were observed within each of the three sites (*r* = 0.68, *p* = 0.02 in Phytoplankton; *r* = 0.95, *p* < 0.0001 in Mangrove; *r* = 0.87, *p* < 0.001 in Seagrass, *n* = 10).

**FIGURE 3:**
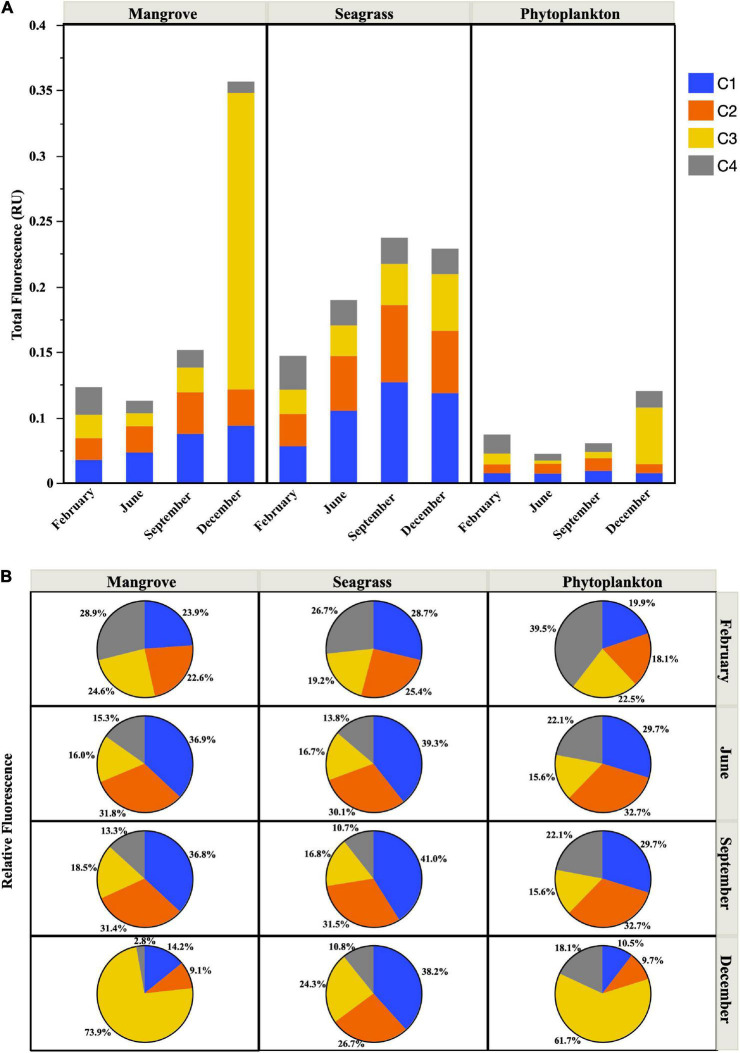
Total fluorescence (Raman Units, RU) **(A)** and relative abundance of the FDOM components C1, C2, C3, and C4 **(B)** at the time of sample collection at the three sites.

The fluorescent properties of DOM showed clear seasonal variations, with the percent contribution of protein-like substances higher in February and December and lower during the warmest months, especially at the Mangrove and Phytoplankton sites (Figures 2J–L). Total FDOM (i.e., the sum of the four fluorescent components detected) also showed higher values at the Seagrass site, followed by the Mangrove site (except in December, where the Mangrove site showed 1.7-fold higher fluorescence values due to a huge increase in the C3 peak, which dominated the fluorescence signal, Figure 3A). The lowest FDOM values were consistently observed at the Phytoplankton site regardless of the season (Figure 3A). In the warmest months sampled (June and September), the protein-like fluorescent components C3 (Tryptophan-like) and C4 (Tyrosine-like) were less abundant than the humic-like components (C1 and C2) at the 3 sites, accounting for less than 40% (Figures 2J–L, 3B). At the Seagrass site, a low contribution of protein-like compounds was observed throughout the year (repeated measurements ANOVA, *p* < 0.005, *n* = 30). A remarkable increase in the relative fluorescence of the C3 component was observed in December, reaching 62 and 74% at the Phytoplankton and Mangrove sites, respectively (Figure 3B). The humic-like fluorescent component C1, corresponding to peak A in Coble (2007), showed consistently higher fluorescence intensities than the other components at the Seagrass site. At the Mangrove and Phytoplankton sites, the fluorescent components showed higher variability (Figure 3B).

### Bacterial Abundances and Physiological Structure

**FIGURE 4:**
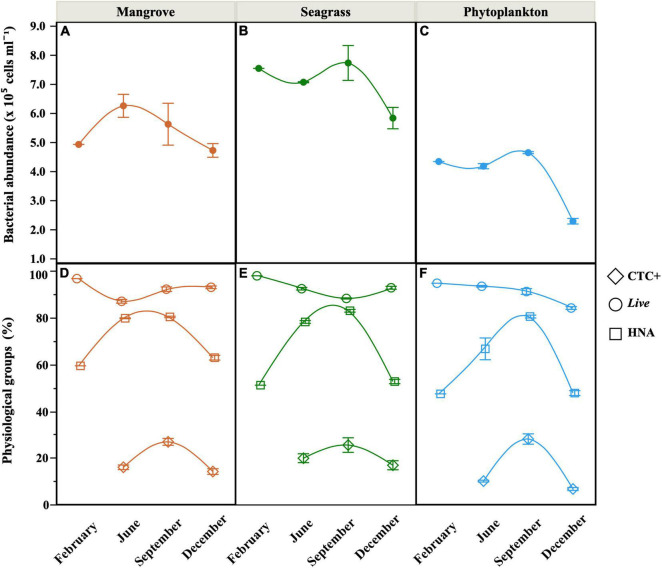
Seasonal distribution of mean (± SE) values of total heterotrophic bacterial abundance **(A–C)**, sum of LNA and HNA cells) and the contribution of *Live*, HNA and CTC+ cells **(D–F)** at the onset of the incubations (unfiltered seawater, equivalent to the Community treatment) at the three sites. Smooth fitting joins site points for clarity.

At the onset of the incubations, total heterotrophic bacterial abundance (i.e., the sum of the LNA and HNA groups) ranged from 2.3 to 7.7 × 10^5^ cells mL^–1^ (Phytoplankton and Seagrass sites, respectively, Figures 4A–C). The Seagrass site showed significantly higher abundances than the other two sites (repeated measurements ANOVA, *p* < 0.001, *n* = 30), while the Phytoplankton site never exceeded 4.5 × 10^5^ cells mL^–1^. HNA cells dominated the community at all sites most of the time, except at the Phytoplankton site in February and December, when %HNA dropped to 47%. A clear seasonal pattern in %HNA emerged at the three sites, with minima in February and December and maxima close to 80% in September (Figures 4D–F). %HNA increased significantly with temperature at the three sites (Supplementary Table 1). The Mangrove site showed a significantly higher contribution of HNA cells (repeated measurements ANOVA, *p* < 0.05, *n* = 30), exceeding on average by 4 and 10% the values of the Seagrass and Phytoplankton sites, respectively. Likewise total abundance, the Seagrass site also showed consistently higher abundance of *Live* cells (maximum of 5.14 × 10^5^ cells mL^–1^), while the lowest abundances of membrane-intact cells were typically found at the Phytoplankton site (minimum of 1.91 × 10^5^ cells mL^–1^). The contribution of *Live* cells (%*Live*) ranged from 84 to 98% (Figures 4D–F) and no significant differences were found between sites. %*Live* cells decreased significantly with increasing temperature only at the Seagrass site (Supplementary Table 1). The abundance of actively respiring cells (CTC+) also peaked at the Seagrass site (maximum 1.93 × 10^5^ cells mL^–1^ in September) with one order of magnitude lower minimum values found at the Phytoplankton site in December (1.50 × 10^4^ cells mL^–1^). The contribution of CTC+ cells followed the same geographical pattern, ranging between 6.6 and 28.0% (Figures 4D–F).

**FIGURE 5:**
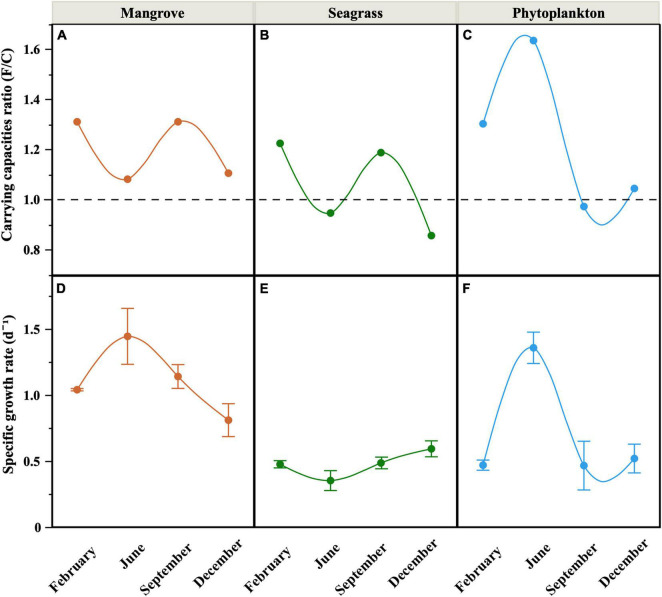
Seasonal distribution of the ratio between the carrying capacities of the Filtered and the Community treatments **(A–C)** and mean (± SE) specific growth rates of heterotrophic bacteria in the Filtered treatment **(D–F)** at the three sites. Smooth fitting joins site points for clarity. Dashed line in **(A–C)** represents the 1 value (i.e., no difference between the maximum bacterial abundances of both treatments).

Total carrying capacity (i.e., the maximum abundance of LNA + HNA cells reached during the incubations) in the Filtered treatment without protistan grazers ranged one order of magnitude, from 3.23 to 10.8 × 10^5^ cells mL^–1^ (Phytoplankton and Seagrass, respectively, data not shown but see Supplementary Table 2 for the individual physiological groups). Minima were observed in December at the three sites, while maximum values were reached in September within the lagoon and in June in offshore waters. Except in June, the Phytoplankton site showed consistently lower carrying capacities than the other two sites, but only significantly lower than the Mangrove site (repeated measurements ANOVA, *p* < 0.05, *n* = 36). The carrying capacity values were generally higher in the Filtered than in the Community treatment, although with some exceptions (Seagrass in June and December and Phytoplankton in September). The corresponding ratio of the Filtered to Community treatment carrying capacities (a surrogate for protistan grazers top–down control of heterotrophic bacterioplankton standing stocks) therefore ranged from 0.86 to 1.64, with the Phytoplankton site showing a different seasonal pattern to both lagoon sites (Figures 5A–C), which seemed to share a common seasonality in grazing pressure likely caused by their proximity (Figure 1B).

### Bacterial Growth Rates

**FIGURE 6:**
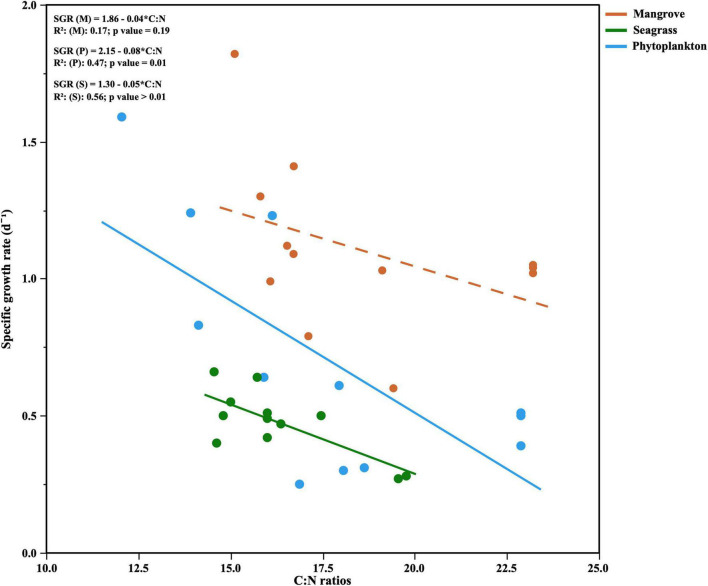
Relationships between the specific growth rates of heterotrophic bacteria and DOM C:N ratios for each site. The OLS linear regression equations and coefficients of determination (*R*^2^) are shown as inserts in the figure. Dashed line represents non significant OLS linear regression.

The growth rates of total heterotrophic bacteria (i.e., LNA + HNA cells) in the Filtered treatments (equivalent to specific growth rates) showed consistently higher values (paired *t*-test, *p* = 0.0013, *n* = 12) than the Community ones (equivalent to net growth rates), similarly to carrying capacities. In the Community treatment we observed mostly positive net growth rates (ranging from 0.06 to 0.85 d^–1^, data not shown), except at the Seagrass site in February, where a decrease rather than an increase in abundance was recorded. In the Filtered treatment, specific growth rates ranged from 0.35 to 1.44 d^–1^ and differed seasonally (Figures 5D–F), with the Mangrove site showing consistently and significantly higher values than the other sites (repeated measurements ANOVA, *p* < 0.01, *n* = 36). Although clear peaks were observed in June for the Mangrove and Phytoplankton sites, no significant correlations were found between specific growth rates and *in situ* temperatures either within individual sites or with all data pooled. Total specific growth rates showed, however, consistently negative relationships with the C:N ratio of DOM, which became significant at the Phytoplankton and Seagrass sites (*r* = –0.69, *p* = 0.0132 and *r* = –0.75, *p* = 0.0054, *n* = 12, respectively, Figure 6).

When it comes to single-cell physiological groups, the specific growth rates of HNA cells ranged from 0.34 (Seagrass in September) to 1.82 d^–1^ (Mangrove in June, Supplementary Figure 1A), while for LNA cells minimum values were observed at the Seagrass site (0.15 d^–1^) and maximum at the Phytoplankton site (1.05 d^–1^), both in June (Supplementary Figure 1B). Similarly to HNA cells, *Live* bacteria also showed minimum and maximum specific growth rates at the Seagrass (0.33 d^–1^) and Mangrove (1.33 d^–1^) sites, respectively (Supplementary Figure 1C). The specific growth rates of both groups shared a similar seasonality (*r* = 0.76, *p* < 0.0001, *n* = 36). Lack of sufficient CTC+ specific growth rate measurements precluded us from apprehending any general pattern besides the fact than values were generally low, never reaching 1 d^–1^ (Supplementary Figure 1D).

### Dissolved Organic Matter Dynamics and Bacterial Growth Efficiencies

Since production and consumption of DOC was concurrently occurring in the Community treatment, drawing any sound conclusion about its dynamics was precluded. On the contrary, with phytoplankton and protistan grazers excluded, DOC dynamics generally showed a decrease with time in the Filtered treatment (Supplementary Figure 2), therefore allowing us to calculate DOC consumption rates. ΔDOC/Δt values ranged from 0.92 to 9.23 μmol C L^–1^ d^–1^ (Table 2). Hereinafter, only results from the Filtered treatment will be presented. The percentage of DOC consumed daily (%DOC) ranged from 0.8 to 9.0% (Seagrass and Mangrove site, respectively) with the Mangrove site showing consistently higher %DOC values than the other sites, but only significantly higher than the Seagrass site (repeated measurements ANOVA, *p* < 0.001, *n* = 27).

**TABLE 2:** Mean (± SE) bacterial biomass increase (ΔBB/Δt), DOC consumption rate (ΔDOC/Δt), and bacterial growth efficiency (BGE) in the Filtered treatment incubations of samples from each month and site.

	Seagrass			Mangrove			Phytoplankton		
	ΔBB/Δt (μmol C L^–1^ d^–1^)	ΔDOC/Δt (μmol C L^–1^ d^–1^)	BGE (%)	ΔBB/Δt (μmol C L^–1^ d^–1^)	ΔDOC/Δt (μmol C L^–1^ d^–1^)	BGE (%)	ΔBB/Δt (μmol C L^–1^ d^–1^)	ΔDOC/Δt (μmol C L^–1^ d^–1^)	BGE (%)
February	0.20 ± 0.03	n.d.	n.d.	0.36 ± 0.03	1.88 ± 0.31	18.0 ± 2.7	0.13 ± 0.01	n.d.	n.d.
June	0.12 ± 0.03	4.48 ± 1.37	3.2 ± 1.0	0.44 ± 0.08	7.77 ± 2.29	6.9 ± 2.2	0.30 ± 0.02	4.27 ± 1.21	8.3 ± 2.1
September	0.24 ± 0.05	3.28 ± 1.15	4.4 ± 1.1	0.41 ± 0.09	9.23 ± 0.07	3.7 ± 1.1	0.15 ± 0.05	5.15 ± 0.81	3.3 ± 1.7
December	0.12 ± 0.01	0.92 ± 0.17	13.2 ± 2.1	0.20 ± 0.04	8.20 ± 2.60	3.1 ± 1.1	0.08 ± 0.01	2.28 ± 1.81	9.8 ± 7.7

*See the text for details. n.d., no data.*

A small but variable part of the amount of DOC consumed by heterotrophic bacteria was used to build up their biomass. This increase in heterotrophic bacterial biomass was minimum in December at the Phytoplankton site (0.08 μmol C L^–1^ d^–1^) and maximum in June in the Mangrove site (0.44 μmol C L^–1^ d^–1^, Table 2), reflecting largely the changes in specific growth rates (Figures 5D–F). Bacterial growth efficiency (BGE), which represents the amount of consumed DOC used for bacterial BP, ranged from 3.1 to 18.0% (Table 2). We observed a significant correlation between the initial DOC concentrations and BGE at the Mangrove (*r* = 0.77, *p* = 0.0081, *n* = 9) and the Seagrass sites (*r* = 0.92, *p* = 0.0012, *n* = 8), but not at the Phytoplankton site. BGE was also significantly and negatively correlated with temperature at the Seagrass site (*r* = –0.77, *p* = 0.0253, *n* = 9) and with all data pooled (*r* = −0.60, *p* = 0.0011, *n* = 27).

The DOM fluorescent components C2 (humic-like) and C4 (protein [Tyrosine]-like) showed consistent consumption during the exponential phase of bacterial growth while C1 (humic-like) and less clearly C3 (protein [Tryptophan]-like) were mostly produced rather than taken up (Supplementary Figure 3). Overall, FDOM components ranged from a consumption rate of −0.0078 R.U. d^–1^ to a production rate of 0.0112 R.U. d^–1^ (C4 and C3, respectively, Supplementary Figure 4A). FDOM dynamics were clearer at the Seagrass site, likely because of its higher initial DOC and FDOM components concentrations regardless of the sampling period (Figures 2D–F and Supplementary Figure 3A). Interestingly, C4 consumption rates increased significantly with nanophytoplankton (chlorophyll *a* size-class) there (*r* = 0.82, *p* = 0.0012, *n* = 9) and at the Mangrove site (*r* = 0.81, *p* = 0.0013, *n* = 9), a relationship that held with all data pooled (*r* = 0.60, *p* = 0.0001, *n* = 26). Bacterial specific growth rate and BGE were both positively correlated to the production rate of C1 at the Seagrass site (*r* = 0.55, *p* = 0.06 and *r* = 0.77, *p* = 0.02, respectively, *n* = 9). BGE was also significantly and positively correlated to the consumption rate of C4 (*r* = 0.71, *p* = 0.05, *n* = 9) there. Consumption of inorganic nutrients was also observed in most incubations (Supplementary Figure 4B). The dynamics of nitrate ranged from a consumption rate of −0.14 μmol L^–1^ d^–1^ to a production rate of 0.32 μmol L^–1^ d^–1^ (Mangrove and Phytoplankton sites, respectively). At the Seagrass site no consumption of nitrate was observed. Changes in nitrite concentration were usually lower than for nitrate, except in February at the Seagrass site where a marked consumption was detected. Phosphate was consistently consumed at the Phytoplankton and Mangrove sites, peaking at −0.054 μmol L^–1^ d^–1^ in September (Mangrove). Phosphate consumption was only observed in September at the Seagrass site, with a maximum production of 0.075 μmol L^–1^ d^–1^ in December.

### Prokaryotic Diversity

**FIGURE 7:**
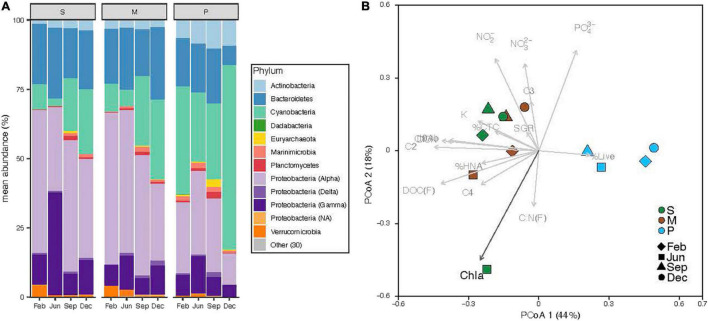
Prokaryotic diversity at the time of sample collection at the three sites (S, Seagrass; M, Mangrove; P, Phytoplankton), showing the relative abundance of sequences belonging to the top 10 most abundant phyla, with Proteobacteria further divided into its major classes **(A)** and distribution of samples according to Principal Coordinates Analysis of Bray–Curtis distances with biological and environmental variables overlaid **(B)**. Black arrows show significant effects (*p* > 0.05).

The analysis of the 16S rRNA gene confirmed that Archaea in surface coastal waters represented a very low fraction of the total abundance of prokaryotes (0.5% ± 0.2%). Overall, the most abundant phyla were Proteobacteria (49%), Cyanobacteria (24%, of which 94% were *Synechococcus* spp.) and Bacteroidetes (19%) (Figure 7A). Although their relative contribution differed between sites, differences at the phylum level were not statistically significant. The Seagrass and Mangrove sites showed fewer Cyanobacteria than the Phytoplankton site (ANOVA, marginally significant differences *p* = 0.055, *n* = 12) and different Proteobacterial groups, mainly in the Alphaproteobacteria class. The Seagrass and Mangrove sites had a higher percentage of the order Rhodospirilalles and Rhodobacteralles, while the Phytoplankton site had higher percentages of the SAR11 clade and Puniceispirillales (Supplementary Figure 5). Overall, the number of ASVs detected at each site was not significantly different (406 ± 33, *n* = 12), nor was the Shannon index (3.9 ± 0.1, *n* = 12). The distribution of samples in a Principal Coordinates Analysis showed the existence of 2 clusters along the PCoA 1 axis (Figure 7B), one gathering Mangrove and Seagrass samples (except June of the latter one) and another separating all Phytoplankton samples, representing the inshore-offshore gradient in the lagoon. Of all the environmental and biological variables considered, only chlorophyll *a* had a significant effect explaining the distribution of the samples.

## Discussion

The quality and quantity of DOM are considered the main factors limiting the growth of heterotrophic bacteria (Church, 2008), with temperature emerging as a secondary factor affecting DOM processing (Apple et al., 2006; Kirchman et al., 2009). While our three sites shared a simil ar seasonality in temperature spanning 10°C (6°C at the Phytoplankton site, Figure 2A), bottom–up control showed clear differences related to the dominant source of DOM present at each location. We have recently suggested that the specific growth rates of heterotrophic bacterioplankton in the central Red Sea are indeed controlled by resource availability rather than by temperature (Silva et al., 2019). However, with only a few exceptions (e.g., Weisse, 1989; Grossart and Simon, 2002; Calleja et al., 2018; García et al., 2018; Silva et al., 2019), the dynamics of heterotrophic bacterioplankton in the Red Sea remain largely unexplored, with the exception of studies conducted mostly in its northern reaches (e.g., Berninger and Wickham, 2005). In particular, their individual physiological status has only been assessed once in a coastal embayment near KAUST (Silva et al., 2019). One of the purposes of this study was to widen the above-mentioned observations by including the first seasonal assessment, in the Red Sea, of bacterial single-cell physiological groups in natural coastal sites each dominated by one of the major DOM sources found in tropical regions (seagrasses, mangroves, and phytoplankton). We must note that our observations were not solely affected by DOM of different origin, but other factors such as different trophic relationships or residence time (longer within the lagoon) may have played a role. Yet, it is worth mentioning that although the bacterial communities from the nearby lagoon sites Mangrove and Seagrass (Figure 1B) were closer in terms of diversity (except Seagrass in June, see Figure 7B), they yielded strong differences in almost all remaining variables (Figures 2, 4–6).

The abundances of heterotrophic bacteria, particularly inside the coastal lagoon (mean ± SE values of 5.4 ± 0.3 at the Mangrove and 7.0 ± 0.4 × 10^5^ cells mL^–1^ at the Seagrass site) were more similar to values previously reported across the Red Sea (Wiebinga et al., 1997; Grossart and Simon, 2002; Kürten et al., 2015), and therefore higher than those observed at KAUST Harbor (1.5–4.8 × 10^5^ cells mL^–1^, Silva et al., 2019). However, the average of the four samples from the Phytoplankton site (3.9 ± 0.5 × 10^5^ cells ml^–1^) was very similar to the annual averages of monthly samplings collected at KAUST Harbor (Silva et al., 2019; Sabbagh et al., 2020) and its seasonality matched that found at the surface of a more offshore station 700 m deep (Al-Otaibi et al., 2020). The almost twofold higher abundances at the Mangrove and Seagrass sites located inside the coastal lagoon compared with both the Phytoplankton site and KAUST Harbor can thus be partially explained by higher resource availability, evidenced in the significantly higher concentrations of DOC and Chl *a* (Table 1). However, the maximum total abundances or carrying capacities across these three new sites were not particularly different from those reached in the shallow waters of KAUST Harbor, with bacterial abundance exceeding 1 million cells mL^–1^ only once at the Seagrass site. Although lower DOM (both DOC and DON) concentrations were consistently found at the Mangrove and Phytoplankton sites (Figures 2D–I), we found generally higher ratios between the carrying capacities of the Filtered and the Community treatment there compared with the Seagrass site, which was even significantly lower than 1 in half of the incubations (Figures 5A–C). Higher DOM concentrations at the Seagrass site would in principle allow for longer, enhanced growth when mortality due to grazing was excluded. One plausible explanation is that top–down control by protistan grazers was generally stronger at the Mangrove and Phytoplankton sites, as previously found in the nearby shallow waters of KAUST Harbor (Silva et al., 2019; Sabbagh et al., 2020). That the carrying capacities reached in the Community treatment were indeed higher than in the Filtered treatment twice (therefore yielding F/C ratios below 1, Figure 5B), points out to a loose top–down control of heterotrophic bacteria by protistan grazers in the vicinity of *Cymodocea serrulata* seagrass meadows on those occasions. The obvious alternative is that bottom-up control was overriding top-down control (Morán et al., 2010), due to the less labile nature of seagrass DOM (reflected for instance in the consistently lower percentages of proteinaceous FDOM, Figures 2J–L), so that bacterial growth was more limited by substrate availability than by protistan grazing. Additionally, this weaker top-down control could be also related to a possible antiprotistan effect of seagrass exudates (Trevathan-Tackett et al., 2015), that would keep the abundance of bacterial predators low in the Community treatment, allowing for a greater effect of additional inputs of DOM from the complete microbial food web (i.e., released by large phytoplankton primary production or leaked via other food web interactions). Interestingly, C:N ratios were similarly high at the three sites (Table 1). The influence of mangroves and seagrass meadows could explain the high C:N ratios within the rather shallow and enclosed lagoon waters: values as high as 33 and 41 have been reported in *A. marina* mangrove sediments in the Red Sea (Saderne et al., 2020), while values over 30 were observed for tropical *C. serrulata* leaves, which increased under high temperatures and low nutrient concentrations (Viana et al., 2020). In the case of the Phytoplankton site outside the lagoon, C:N ratios higher than the nearby KAUST Harbor waters (Silva et al., 2019) can be explained by mixing with lagoon water or to its more oligotrophic nature, evidenced in the comparatively lower concentrations of inorganic nitrogen (Table 1).

Regarding the individual physiological structure, in spite of the fact that we had only four snapshots of the seasonal cycle, the three sites along the inshore-offshore lagoon gradient showed patterns similar to those recently reported for the as well shallow waters of KAUST Harbor (Silva et al., 2019). The contribution of HNA cells was notably higher than normally found in tropical oligotrophic waters (e.g., Gasol et al., 2009; Baltar et al., 2012; Bock et al., 2018), even at the more nutrient limited Phytoplankton site (>40%, Figures 4D–F). HNA cells are normally made up of copiotrophic and more active taxa (Lebaron et al., 2001; Morán et al., 2007), such as Rhodobacterales or Bacteroidetes (Vila-Costa et al., 2012; Mayali et al., 2014), and consequently HNA clearly outgrew LNA cells during all incubations as previously observed (Huete-Stauffer et al., 2015; Silva et al., 2019; Morán et al., 2020). The contribution of CTC+ cells was also relatively high in the three ecosystems (5–32%, Figures 4D–F), confirming prior findings (Silva et al., 2019). However, although the physiological structure would point out to a large growth potential, we did not observe particularly high specific growth rates (Figures 5D–F). Indeed, with exception of the Mangrove site and once at the Phytoplankton site, total heterotrophic bacteria growth rates were notably lower than the values found at KAUST Harbor in 2016 (mean 1.26 d^–1^, Silva et al., 2019). Accordingly, the specific growth rates of the *Live* and CTC+ groups (Supplementary Figure 1) were also on average twofold lower than at KAUST Harbor (Silva et al., 2019). Interestingly, the specific growth rates of the total community at the three sites were similar to values obtained in incubations performed in the Great Barrier Reef (Australia) with coastal heterotrophic bacterioplankton previously enriched with DOM from seagrass and mangrove leachates as well as glucose on top of the naturally occurring values (0.24–1.93 d^–1^, Morán et al., 2020). Also, the higher growth rates found at the Mangrove site follow the same response as in Australian waters, where mangrove enriched-DOM induced a much higher (>4-fold) response in specific growth rates than seagrass enriched-DOM (Morán et al., 2020). Remarkably, although the seagrass and mangrove species differed between the two regions (*Cymodocea serrulata* and *Avicennia marina* in the Red Sea vs. *Halodule uninervis* and *Rhizophora stylosa* in the Great Barrier Reef waters, Morán et al., 2020), the single-cell physiological groups showed similar responses. In our experiments, the expected enhancement in bacterial growth rates with temperature (Brown et al., 2004) was only found for *Live* cells in the Mangrove and Phytoplankton site and for the LNA cells in the Phytoplankton-dominated site (Supplementary Table 1). The absence of a clear temperature dependence, similar to nearby KAUST Harbor observations (Silva et al., 2019), together with the low specific growth rates found at the Phytoplankton (except in June) and Seagrass sites suggest a strong bottom–up limitation of bacterial activity, as frequent in other tropical and subtropical waters (Morán et al., 2017). Moreover, we also observed consumption of inorganic nutrients in around 70% of the incubations, including the Seagrass site (Supplementary Figure 4B), suggesting that phytoplankton and macrophyte DOM substrates might not suffice in supplying the necessary nutrients for bacterial growth in Red Sea coastal ecosystems. The analysis of fluorescent DOM, in particular the percentage of protein-like components, also hints at the hypothesis that variations in DOM quality were able to partially explain our results. For instance, when the community was dominated by cyanobacteria (Phytoplankton site, Figure 7A), the total amount of FDOM was lower than at the Seagrass and Mangrove sites (Figure 3A). In December there was a marked decrease in the relative abundance of alphaproteobacteria at all sites (Figure 7A) associated with an increase in the relative abundance of the C3 Tryptophan-like component (Figure 3B), which was proven to be much less labile in the incubations than the C4 Tyrosine-like one (Supplementary Figure 3). Moreover, the relationships of C4 consumption rates with the various chlorophyll *a* size-classes suggest that nanophytoplankton, present at all sites, entails a source of labile Tyrosine-like component, particularly in the mangrove and seagrass dominated systems. The overall negative correlation between total bacterial specific growth rates and DOM C:N ratios (Figure 6), in particular at the Phytoplankton and Seagrass sites, suggest nitrogen-rich DOM enhances bacterial growth by increasing the efficiency at which dissolved compounds are assimilated and transformed into new bacterial biomass. In that regard, degradation rates of DON are often higher than DOC (Hopkinson et al., 2002; Lønborg et al., 2009b). Indeed, the high growth rates observed in June at the Phytoplankton site were associated with the maximum DON concentration found at this site (Figure 2I). It is interesting to note that for the gradient of overlapping C:N molar ratios at the 3 sites (i.e., 15–20), specific growth rates at the Seagrass site were 1.2–2.4-fold consistently lower than at the other two sites, including the much more oligotrophic Phytoplankton site (Figures 5D–F and Table 1). Even though the Seagrass incubations were apparently characterized by the highest resource availability, as indicated by higher DOC and DON concentrations and generally also total FDOM intensity (Figures 2D–I, 3A and Table 1), they consistently showed the lowest potential of heterotrophic bacterial growth, as well as the lowest proportion in the most labile fluorescent component (Tyrosine-like, C4) and a conversely high proportion in humic material (C1 and C2) year-round (Figure 3B). The relatively low bacterial specific and net growth rates together with the low ratios between the Filtered and Community treatment carrying capacities at the Seagrass site could be due either to the lack of some key micronutrient or vitamin, or to the lower DOC bioavailability of the seagrass exudates (Table 2, Lønborg et al., 2019). We cannot completely exclude a possible antibacterial activity in the seagrass exudates, as has been already described for several seagrass species, including *Cymodocea serrulata* (Kumar et al., 2008; Supaphon et al., 2013), the species dominating our Seagrass site. If present, the antimicrobial effect of seagrass DOM seemed to affect only bacterial numbers and rates, since the overall community composition of prokaryotes was quite similar at the two sites dominated by macrophytes (Figure 7). In contrast, both lagoon locations differed notably in their prokaryotic composition from the Phytoplankton site, which showed a community more similar to that expected in the more oligotrophic open ocean (Biers et al., 2009). The large differences between Mangrove and Seagrass specific growth rates (more than double on average at the former site, Figures 5D,E) would thus be driven by DOM composition, as suggested by differences in the contribution of protein-like and humic-like fluorescent compounds, and not as much by absolute DOM concentration or bacterial community composition.

Bacterial growth efficiency (i.e., the ratio of heterotrophic bacterioplankton biomass produced per unit carbon consumed) has proven useful to understand the role of bacteria in marine food webs (del Giorgio and Cole, 1998; Reinthaler and Herndl, 2005; Alonso-Sáez et al., 2008; Lønborg et al., 2011). In spite of the relatively high DOC and DON concentrations, in particular inside the lagoon (Figure 2B), our BGE values were generally low (3.1–17.9%), well in agreement with those reported by Silva et al. (2019), clearly ascribing the coastal ecosystems of the central Red Sea to the oligotrophic type (del Giorgio and Cole, 1998; Robinson, 2008). We unexpectedly observed significant, negative correlations between BGE and initial DOC concentrations at the Mangrove and Seagrass sites. This negative relationship supports the contention that bulk measurements of DOC do not consistently inform us about its biological lability (Carlson and Hansell, 2014). On the other side, the negative correlation between DOM C:N ratios and specific growth rates found in this study together with the positive correlation between BGE and DON concentration reported for the nearby KAUST Harbor (Silva et al., 2019) highlight the importance of nitrogen in organic compounds for bacterial biomass build-up. The importance of DOM composition in determining BGE is evidenced by the highly consistent consumption of the FDOM protein-like component C4 (consumed in 92% of the incubations, Supplementary Figure 4A) and the positive correlation of BGE with its initial fluorescence and consumption rate. There is mounting evidence that fluorescent protein-like substances are being selectively taken up by heterotrophic prokaryotes throughout the entire water column of the central Red Sea, fostering their growth (Silva et al., 2019; Morán et al., 2022).

Even though individually it was only significant at the Seagrass site, a negative correlation was also found between BGE and temperature. This negative correlation is in agreement with the study by Rivkin and Legendre (2001), later confirmed in other works (Apple et al., 2006; Hall and Cotner, 2007), but is exactly the opposite of what we found in KAUST Harbor waters (Silva et al., 2019). Since production rates of C1 and consumption rates of C4 were also higher in February and December (Supplementary Figure 4), our study suggests a better performance of heterotrophic bacteria from and around Al Monseini lagoon at temperatures below 30°C. As argued in López-Urrutia and Morán (2007), relationships between BGE and temperature might be strongly mediated by the trophic status of the system, specifically in oligotrophic and warm regions, where resource limitation may keep bacterial respiration at the expense of bacterial production.

In conclusion, the location along the inshore-offshore gradient of a coastal Red Sea lagoon, aimed at covering the major microphyte and macrophyte sources of DOM in tropical shallow ecosystems, affected differently and coherently the specific growth rates of heterotrophic bacteria, while its effect on their maximum abundances was not that clear. In spite of high DOM concentrations, particularly in waters overlying seagrass meadows, bacterial specific growth rates were much lower than expected. This limited, seasonally invariant bacterial response suggests other nutrients may be limiting, reinforcing the hypothesis of bottom–up control of bacterial activity in shallow waters of the central Red Sea, but also the presence of possible antibacterial or antiprotistan compounds in seagrass meadows. Considering the three sites together, we have found additional evidence that DOM composition affects the specific growth rates and carbon conversion efficiencies of heterotrophic bacteria in shallow tropical waters more than bacterial community composition. In parallel, heterotrophic bacteria standing stocks, in particular at the Mangrove site year-round and the Phytoplankton site during the first half of the year (Figures 5A,C), seemed to be top–down controlled by the activity of protistan grazers. Seasonality in top–down control by switching from protistan grazing to viral lysis as the dominant process has been reported for the nearby waters of KAUST Harbor (Sabbagh et al., 2020). The role of temperature on heterotrophic bacteria growth rates and carrying capacities in these tropical waters was either non-existent or negative, especially under resource-limited conditions.

## Data Availability Statement

The raw 16S rRNA gene amplicon data can be found in the European Nucleotide Archive, project number PRJEB49205. The rest of the data are included in the Supplementary Material.

## Author Contributions

LS performed the experiments, analyzed the data, prepared Figures 1–5 and tables, and wrote the manuscript. MLIC performed the DOM analysis, contributed to the interpretation of results and writing. TMH-S performed the 16S rRNA analysis, prepared Figure 6, and contributed to the interpretation of results and writing. SI processed the DOM samples. MIA processed the 16S rRNA samples. MV helped with the experimental setup and sample collection. XAGM conceived the research, contributed to data analysis and the interpretation of results and writing. All the authors contributed to the article and approved the submitted version.

## Conflict of Interest

The authors declare that the research was conducted in the absence of any commercial or financial relationships that could be construed as a potential conflict of interest.

## Publisher’s Note

All claims expressed in this article are solely those of the authors and do not necessarily represent those of their affiliated organizations, or those of the publisher, the editors and the reviewers. Any product that may be evaluated in this article, or claim that may be made by its manufacturer, is not guaranteed or endorsed by the publisher.
