# Supplementary material for: Heterotrophic Bacterioplankton Growth and Physiological Properties in Red Sea Tropical Shallow Ecosystems With Different Dissolved Organic Matter Sources
Source: Front Microbiol. 2022 Jan 3;12:784325. doi: 10.3389/fmicb.2021.784325 (PMC8762102; doi:10.3389/fmicb.2021.784325)
Supplement: Supplementary file 1 [file Data_Sheet_1.pdf]

Supplementary Material

Frontiers in Microbiology 12: 784325

Heterotrophic bacterioplankton growth and physiological properties in Red Sea tropical shallow ecosystems with different DOM sources

Luis Silva[1]*, Maria Ll. Calleja[1,2], Tamara Huete-Stauffer[1], Snjezana Ivetic[1], Mohd I. Ansari[1,3], Miguel Viegas[1], Xosé Anxelu G. Morán1[1,4]*

[1] Division of Biological and Environmental Sciences and Engineering, Red Sea Research Center, King Abdullah University of Science and Technology, Thuwal, Saudi Arabia

[2] Department of Climate Geochemistry, Max Planck Institute for Chemistry (MPIC), Mainz, Germany;

[3] Department of Biosciences, Integral University, Lucknow, India

[4] Centro Oceanográfico de Gijón/Xixón (IEO, CSIC), Gijón/Xixón, Spain

*** Correspondence:**

Luis Silva: luis.ribeirodasilva@kaust.edu.sa
Xosé Anxelu G. Morán xelu.moran@kaust.edu.sa, xelu.moran@ieo.es

Running title: Tropical bacteria growth and DOM

Supplementary Table 1. Pearson correlation coefficients between *in situ* temperature and the four single-cell physiological groups absolute (cells mL^{-1}) and relative (%) abundances at the onset of the experiments, carrying capacities (K, cells mL^{-1}) and specific growth rates (μ, d^{-1}). **, p<0.01; *, p<0.05; ns, non-significant. HNA, high nucleic acid bacteria; LNA. low nucleic acid bacteria; *Live*, membrane-intact cells; CTC+, actively respiring cells.

Site	Physiological group	Absolute abundance	Relative abundance	K	μ
Seagrass	HNA	0.86**	0.94**	0.79	-0.71**
	LNA	-0.95**	ns	ns	ns
	Live	0.84**	-0.90**	0.90**	-0.59*
	CTC+	0.92**	0.71*	0.88**	-0.92*
Mangrove	HNA	0.69*	0.91**		ns
	LNA	-0.87**	ns	-0.76**	ns
	Live	ns	ns	0.78**	0.68*
	CTC+	0.95**	0.87**	0.91**	ns
Phytoplankton	HNA	0.69*	0.83**	0.68*	ns
	LNA	-0.83**	ns	-0.61*	0.67*
	Live	ns	ns	0.64*	0.69*
	CTC+	0.97**	0.95**	0.8**	-0.93**

Supplementary Table 2. Mean (± SE) carrying capacities (x 10^5 cells ml^{-1}) in the Filtered treatment of the four single-cell physiological groups. HNA, high nucleic acid bacteria; LNA. low nucleic acid bacteria; *Live*, membrane-intact cells; CTC+, actively respiring cells.

	**Seagrass**				**Mangrove**				**Phytoplankton**			
	**HNA**	**LNA**	***Live***	**CTC+**	**HNA**	**LNA**	***Live***	**CTC+**	**HNA**	**LNA**	***Live***	**CTC+**
February	4.85 ± 0.11	4.37 ± 0.05	4.50 ± 0.32	n.d.	6.55 ± 0.47	2.34 ± 0.21	4.83 ± 0.22	n.d.	3.60 ± 0.13	2.92 ± 0.17	2.45 ± 0.22	n.d.
June	5.34 ± 0.24	1.57 ± 0.10	5.43 ± 0.18	1.32 ± 0.01	7.14 ± 0.32	1.48 ± 0.05	7.43 ± 0.03	1.23 ± 0.03	6.92 ± 0.26	2.59 ± 0.03	8.86 ± 0.17	0.91 ± 0.04
September	8.04 ± 0.20	2.74 ± 0.39	6.93 ± 0.20	1.93 ± 0.33	8.24 ± 0.26	1.44 ± 0.03	6.90 ± 0.21	1.28 ± 0.02	7.30 ± 0.19	1.20 ± 0.04	5.35 ± 0.15	1.25 ± 0.21
December	0.74 ± 0.12	2.86 ± 0.27	5.02 ± 0.08	0.69 ± 0.06	4.02 ± 0.34	2.67 ± 0.14	6.76 ± 0.22	0.80 ± 0.02	2.18 ± 0.03	1.05 ± 0.09	4.85 ± 0.56	0.67 ± 0.02

Supplementary Figure 1. Seasonal distribution of mean (± SE) values of the specific growth rates (i.e., Filtered treatment) of HNA (**A**), LNA (**B**), *Live* (**C**) and CTC+ cells (**D**) at the three sites. Note that CTC+ cells specific growth rates in September could not be calculated because no growth was observed. Smooth fitting joins complete site points for clarity. Color coding: blue, Phytoplankton; orange, Mangrove; green, Seagrass.

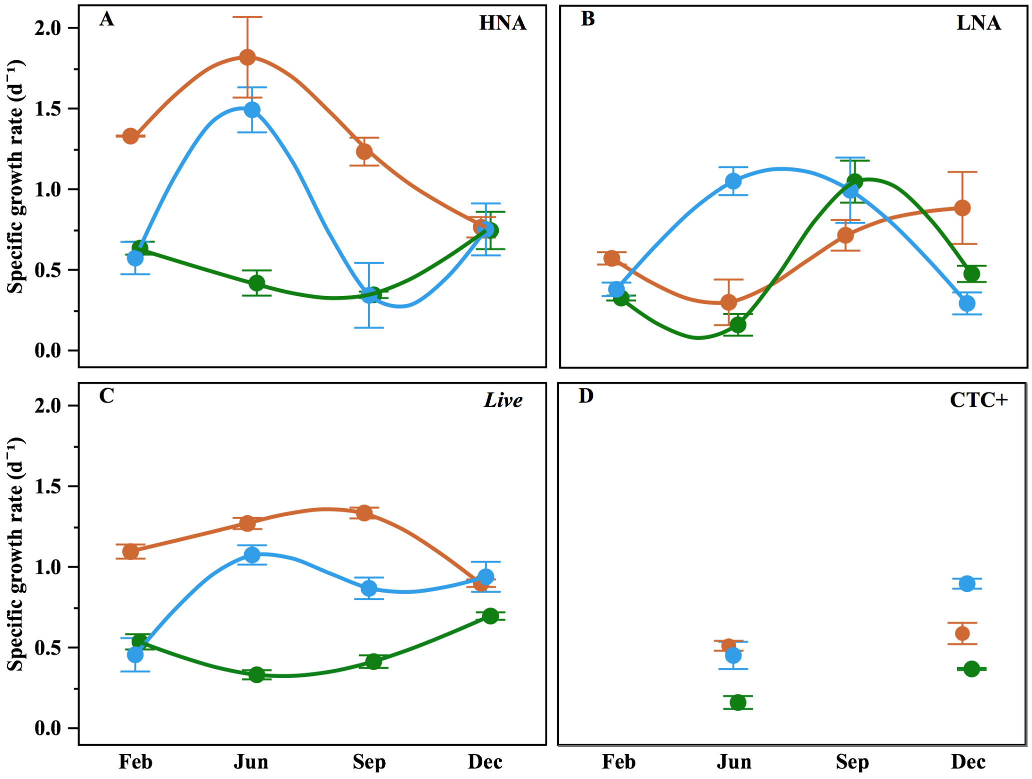

Supplementary Figure 2. Temporal changes in DOC concentration in the Filtered treatment incubations of samples from each month and site. Color coding: blue, Phytoplankton; orange, Mangrove; green, Seagrass.

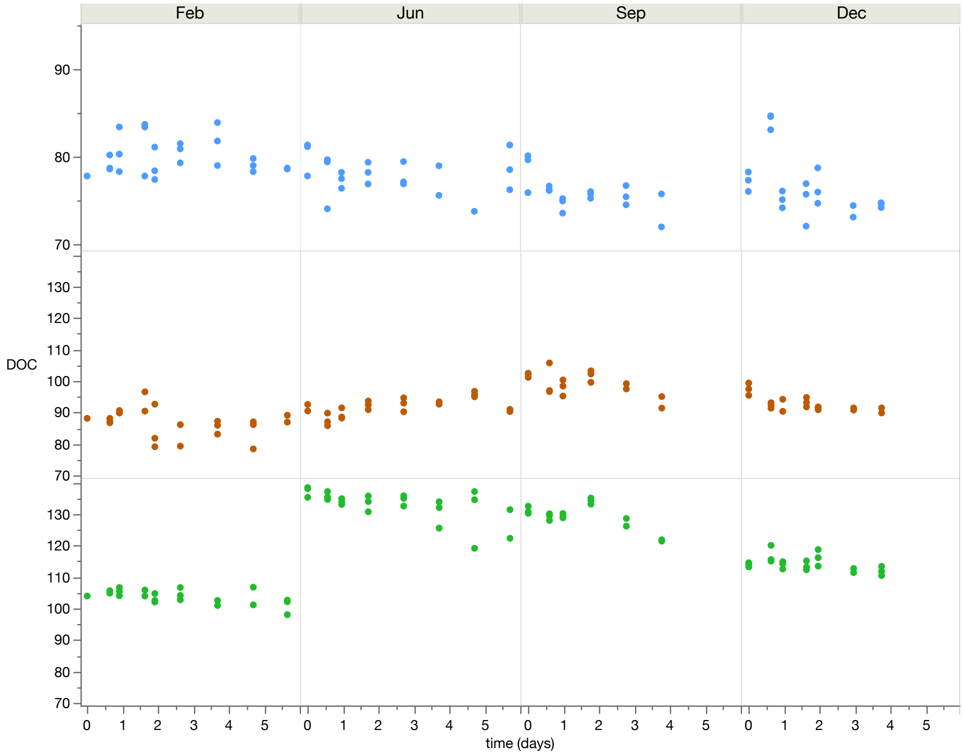

Supplementary Figure 3. Temporal changes in fluorescence (relative units) of the humic-like fluorescent DOM components C1 and C2 (**A**) and the protein-like fluorescent DOM components C3 and C4 (**B**) in the Filtered treatment incubations of samples from each month and site. Color coding: blue, Phytoplankton; orange, Mangrove; green, Seagrass.

A

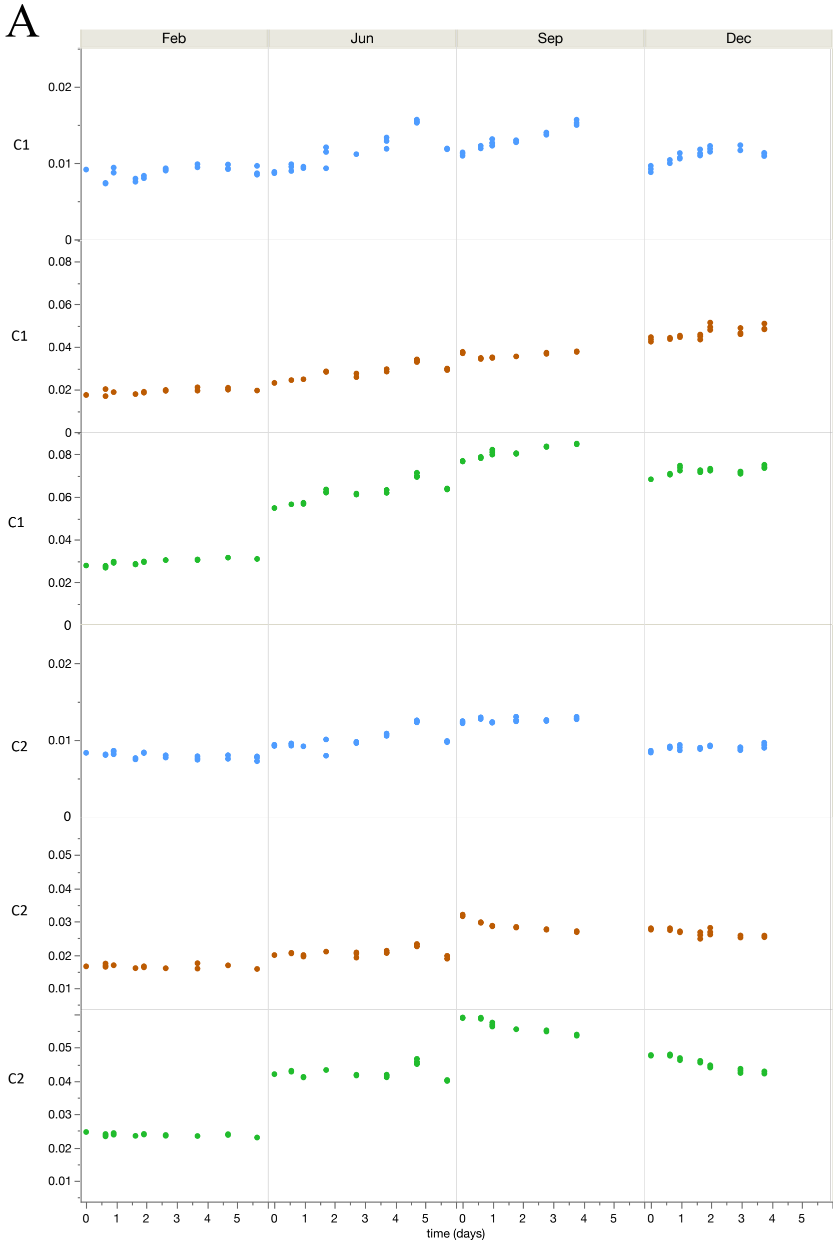

Supplementary Figure 3 (continued)

B

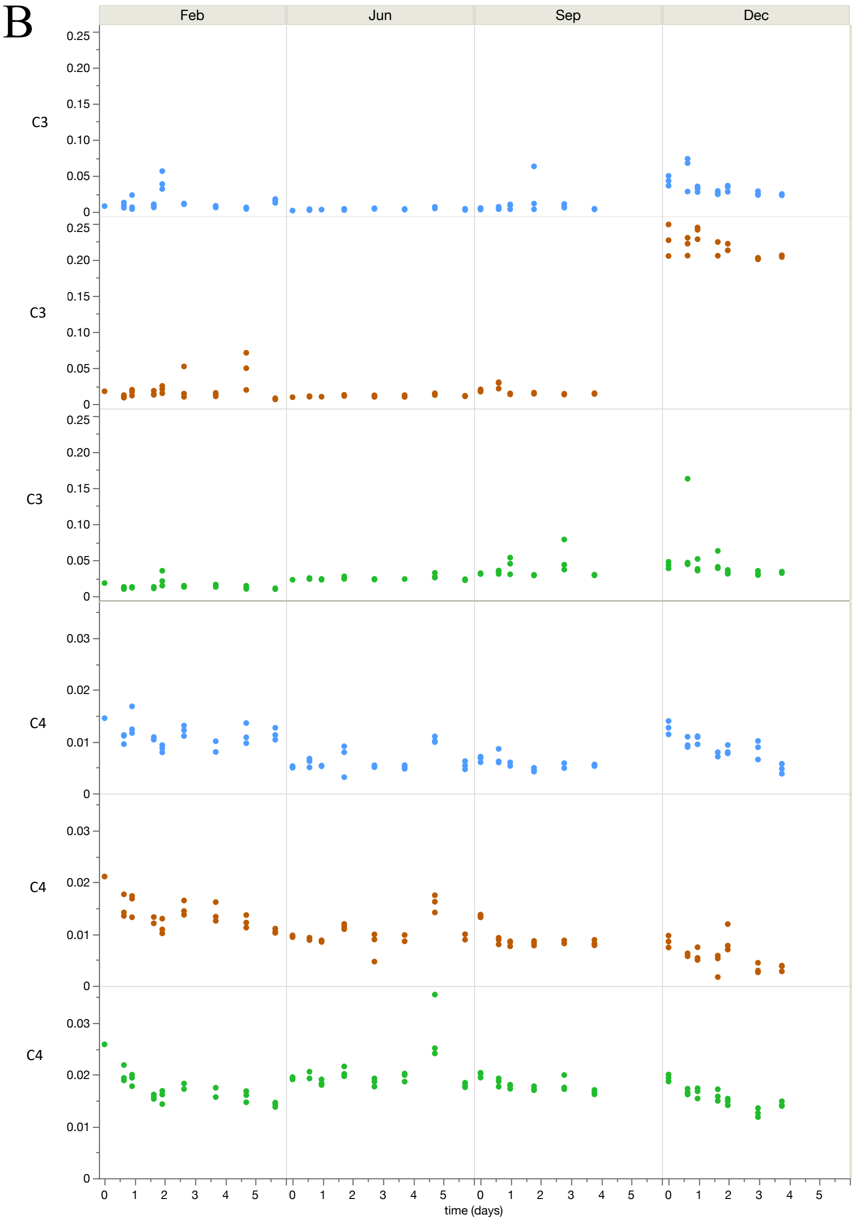

Supplementary Figure 4. Seasonal distribution of mean (± SE) production (i.e., values>0) and consumption (i.e., values<0) rates of the fluorescent DOM components C1, C2, C3 and C4 (R.U. d^{-1}, **A**) and the inorganic nutrients nitrate, nitrite and phosphate ($\mu mol\ L^{-1}\ d^{-1}$, **B**) in the Filtered treatment incubations of the three sites. No phosphate dynamics were available in February. See the text for details.

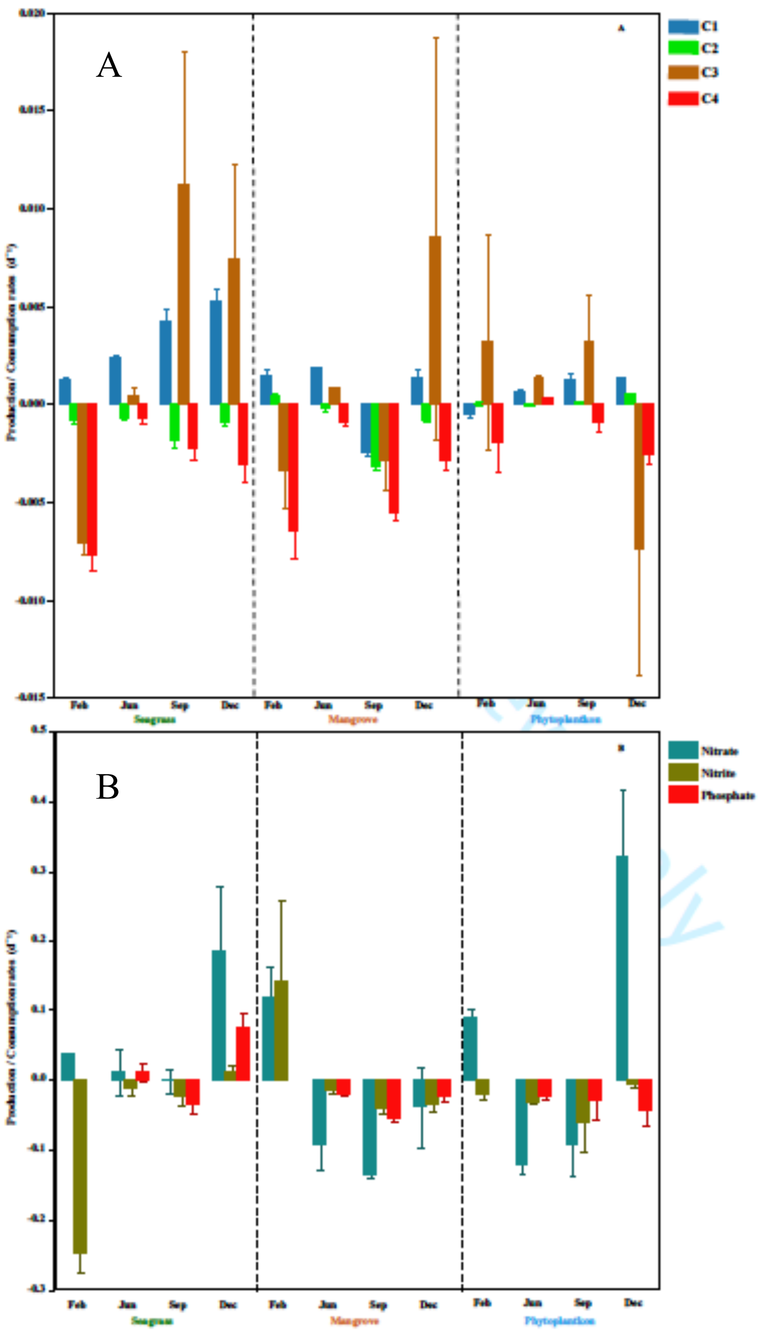

Supplementary Figure 5. Contribution of the different orders to total Alphaproteobacteria at the time of sample collection at the three sites (S, Seagrass; M, Mangrove; P, Phytoplankton) for each month (bar chart) and averaged for each system for the most relevant orders (table).

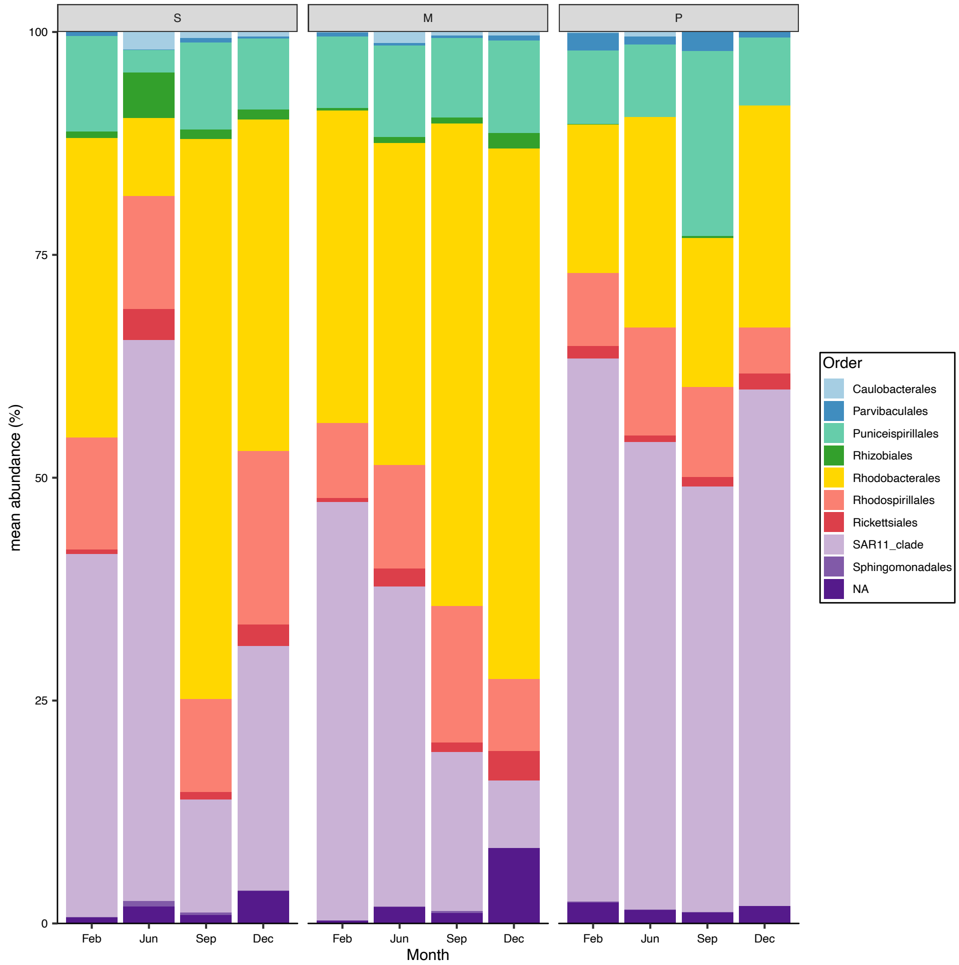

Alphaproteobacteria Order (%)	**S**	**M**	**P**
SAR11 clade	35 ± 10	27 ± 9	55 ± 3
Puniceispirillales	8 ± 2	9 ± 1	11 ± 3
Rhodospirilalles	14 ± 2	11 ± 2	9 ± 1
Rhodobacteralles	35 ± 11	46 ± 6	20 ± 2

Month	Station	Temp	Tot abu t0	LNA abu t0	HNA abu t0	%HNA	*Live* abu t0	%Live	CTC+ abu t0	%CTC+	µ Comm	µ Filt	µ LNA	µ HNA	µ *Live*	µ CTC+	K Comm	K Filt	K F/C	K LNA	K HNA
February	Phytoplankton	25.1	4.33E+05	2.05E+05	2.28E+05	47.3	2.00E+05	94.7			0.13	0.50	0.29	0.72	0.64		4.98E+05	6.31E+05	1.30	2.58E+05	3.73E+05
February	Phytoplankton	25.1	4.33E+05	2.05E+05	2.28E+05	47.3	2.00E+05	94.7			0.17	0.39	0.40	0.38	0.44		5.09E+05	6.49E+05	1.30	3.15E+05	3.34E+05
February	Phytoplankton	25.1	4.33E+05	2.05E+05	2.28E+05	47.3	2.00E+05	94.7			0.10	0.51	0.43	0.61	0.28		4.94E+05	6.76E+05	1.30	3.02E+05	3.74E+05
February	Mangrove	22.4	4.92E+05	2.92E+05	2.00E+05	59.3	2.78E+05	96.7			0.23	1.05	0.61	1.32	1.15		6.62E+05	9.82E+05	1.31	2.71E+05	7.10E+05
February	Mangrove	22.4	4.92E+05	2.92E+05	2.00E+05	59.3	2.78E+05	96.7			0.38	1.02	0.49	1.33	1.01		6.51E+05	7.62E+05	1.31	2.00E+05	5.62E+05
February	Mangrove	22.4	4.92E+05	2.92E+05	2.00E+05	59.3	2.78E+05	96.7			0.06	1.04	0.60	1.33	1.12		7.21E+05	9.23E+05	1.31	2.32E+05	6.91E+05
February	Seagrass	22.4	7.54E+05	3.85E+05	3.69E+05	51.1	4.28E+05	97.9				0.49	0.31	0.67	0.48		7.54E+05		1.23		
February	Seagrass	22.4	7.54E+05	3.85E+05	3.69E+05	51.1	4.28E+05	97.9				0.42	0.30	0.55	0.49		7.54E+05	9.40E+05	1.23	4.43E+05	4.98E+05
February	Seagrass	22.4	7.54E+05	3.85E+05	3.69E+05	51.1	4.28E+05	97.9				0.51	0.35	0.67	0.63		7.54E+05	9.07E+05	1.23	4.32E+05	4.75E+05
June	Phytoplankton	29.8	4.13E+05	2.69E+05	1.44E+05	65.1	4.77E+05	94.1	3.92E+04	9.5	0.36	1.24	0.95	1.36	0.96	0.36	6.29E+05	1.00E+06	1.64	2.61E+05	7.41E+05
June	Phytoplankton	29.8	4.34E+05	3.27E+05	1.07E+05	75.3	3.92E+05	92.9	4.20E+04	9.7	0.47	1.23	0.97	1.34	1.08	0.36	5.49E+05	9.08E+05	1.64	2.53E+05	6.56E+05
June	Phytoplankton	29.8	4.04E+05	2.40E+05	1.64E+05	59.4	3.96E+05	93.3	4.34E+04	10.7	0.32	1.59	1.22	1.77	1.17	0.62	5.67E+05	9.42E+05	1.64	2.64E+05	6.78E+05
June	Mangrove	30.0	5.68E+05	4.51E+05	1.17E+05	79.4	4.64E+05	88.6	9.87E+04	17.4	0.39	1.41	0.53	1.69	1.34	0.46	7.46E+05	9.37E+05	1.08	1.58E+05	7.79E+05
June	Mangrove	30.0	6.06E+05	4.85E+05	1.21E+05	80.0	4.94E+05	86.4	9.86E+04	16.3	0.66	1.82	0.31	2.30	1.23	0.49	8.42E+05	8.27E+05	1.08	1.48E+05	6.79E+05
June	Mangrove	30.0	7.01E+05	5.59E+05	1.42E+05	79.7	4.75E+05	86.1	9.85E+04	14.1	0.28	1.09	0.04	1.46	1.23	0.57	8.05E+05	8.24E+05	1.08	1.39E+05	6.85E+05
June	Seagrass	29.4	7.10E+05	5.63E+05	1.47E+05	79.3	5.14E+05	93.5	1.14E+05	16.1	0.08	0.27	0.05	0.34	0.37	0.10	6.66E+05	6.63E+05	0.95	1.48E+05	5.15E+05
June	Seagrass	29.4	7.04E+05	5.47E+05	1.57E+05	77.7	5.12E+05	91.7	1.48E+05	21.0	0.06	0.50	0.28	0.57	0.27	0.12	7.61E+05	7.58E+05	0.95	1.77E+05	5.81E+05
June	Seagrass	29.4	7.05E+05	5.46E+05	1.59E+05	77.4	5.11E+05	92.0	1.57E+05	22.3	0.04	0.28	0.13	0.33	0.33	0.23	7.62E+05	6.51E+05	0.95	1.46E+05	5.05E+05
September	Phytoplankton	31.7	4.58E+05	3.69E+05	8.91E+04	80.6	3.21E+05	93.5	1.47E+05	32.1	0.42	0.83	1.39	0.74	0.98		1.02E+06	8.26E+05	0.97	1.12E+05	7.14E+05
September	Phytoplankton	31.7	4.63E+05	3.77E+05	8.57E+04	81.5	2.66E+05	90.7	1.27E+05	27.4	0.32	0.25	0.73	0.13	0.86		8.22E+05	8.92E+05	0.97	1.25E+05	7.67E+05
September	Phytoplankton	31.7	4.70E+05	3.73E+05	9.70E+04	79.4	2.71E+05	89.4	1.15E+05	24.5	0.17	0.31	0.85	0.14	0.75		7.82E+05	8.31E+05	0.97	1.22E+05	7.09E+05
September	Mangrove	33.3	4.25E+05	3.43E+05	8.19E+04	80.7	3.66E+05	94.1	1.25E+05	29.4	0.56	1.30	0.90	1.38	1.33		7.44E+05	1.02E+06	1.31	1.49E+05	8.72E+05
September	Mangrove	33.3	6.70E+05	5.34E+05	1.36E+05	79.7	3.99E+05	90.9	1.63E+05	24.3	0.07	1.12	0.60	1.23	1.27		8.02E+05	9.60E+05	1.31	1.42E+05	8.18E+05
September	Mangrove	33.3	5.90E+05	4.75E+05	1.15E+05	80.5	3.94E+05	91.5	1.57E+05	26.6	0.26	0.99	0.63	1.08	1.39		6.68E+05	9.21E+05	1.31	1.40E+05	7.81E+05
September	Seagrass	32.7	7.61E+05	6.24E+05	1.37E+05	82.0	5.08E+05	88.5	1.91E+05	25.1	0.27	0.55	1.28	0.36	0.47		8.94E+05	1.09E+06	1.19	3.08E+05	7.85E+05
September	Seagrass	32.7	8.82E+05	7.41E+05	1.41E+05	84.0	5.38E+05	87.7	1.78E+05	20.2		0.50	1.02	0.36	0.34		8.67E+05	1.16E+06	1.19	3.17E+05	8.43E+05
September	Seagrass	32.7	6.75E+05	5.59E+05	1.16E+05	82.8	4.95E+05	88.5	2.09E+05	31.0	0.59	0.40	0.83	0.30	0.41		9.62E+05	9.81E+05	1.19	1.96E+05	7.85E+05
December	Phytoplankton	28.6	2.27E+05	1.13E+05	1.14E+05	49.8	1.98E+05	83.8	1.23E+04	5.4	0.12	0.61	0.36	0.85	1.12	0.83	2.73E+05	3.10E+05	1.05	1.05E+05	2.05E+05
December	Phytoplankton	28.6	2.44E+05	1.14E+05	1.30E+05	46.7	1.90E+05	85.2	1.68E+04	6.9	0.04	0.64	0.35	0.96	0.89	0.92	3.38E+05	4.01E+05	1.05	1.21E+05	2.80E+05
December	Phytoplankton	28.6	2.11E+05	9.84E+04	1.13E+05	46.5	1.85E+05	83.3	1.59E+04	7.5	0.30	0.30	0.15	0.43	0.80	0.92	3.15E+05	2.58E+05	1.05	8.93E+04	1.68E+05
December	Mangrove	26.4	5.09E+05	3.11E+05	1.98E+05	61.1	3.73E+05	94.1	6.08E+04	11.9	0.01	1.03	1.29	0.87	0.90	0.53	5.41E+05	7.63E+05	1.11	2.94E+05	4.68E+05
December	Mangrove	26.4	4.77E+05	3.08E+05	1.69E+05	64.6	3.84E+05	92.7	6.73E+04	14.1	0.56	0.79	0.83	0.76	0.85	0.50	5.79E+05	6.33E+05	1.11	2.58E+05	3.75E+05
December	Mangrove	26.4	4.28E+05	2.69E+05	1.59E+05	62.9	4.31E+05	92.2	6.91E+04	16.1	0.94	0.60	0.52	0.65	0.93	0.71	6.15E+05	6.10E+05	1.11	2.48E+05	3.62E+05
December	Seagrass	25.5	6.04E+05	3.29E+05	2.75E+05	54.5	4.54E+05	91.7	1.20E+05	19.9	1.24	0.66	0.43	0.93	0.68	0.37	8.24E+05	5.46E+05	0.86	2.59E+05	2.87E+05
December	Seagrass	25.5	6.33E+05	3.29E+05	3.04E+05	52.0	4.64E+05	93.9	8.35E+04	13.2	0.07	0.47	0.41	0.53	0.73		6.44E+05	6.00E+05	0.86	2.57E+05	3.43E+05
December	Seagrass	25.5	5.11E+05	2.63E+05	2.48E+05	51.5	4.08E+05	92.8	8.82E+04	17.3	1.24	0.64	0.57	0.76	0.65	0.36	6.98E+05	7.57E+05	0.86	3.41E+05	4.17E+05

K *Live*	K CTC+	DOC	DON	C:N	pChl *a*	nChl *a*	mChl *a*	Chl *a*	BGE	NO_2^-	NO_3^-	PO_4^{-3}	NO_2^- c.r.	NO_3^- c.r.	PO_4^{-3} c.r.	DON c.r	C1	C2	C3	C4	FDOM	%protein	C1 c.r.	C2 c.r.	C3 c.r.	C4 c.r.
2.81E+05		77.8	3.4	22.9	0.11	0.02	0.01	0.14		0.05	0.32		-0.013	0.094		-0.118	0.007	0.007	0.008	0.015	0.037	62.0	-0.001	0.000	-0.004	-0.004
2.49E+05		77.8	3.4	22.9	0.11	0.02	0.01	0.14		0.05	0.32		-0.032	0.107		1.243	0.007	0.007	0.008	0.015	0.037	62.0	0.000	0.000	0.000	-0.003
2.05E+05		77.8	3.4	22.9	0.11	0.02	0.01	0.14		0.05	0.32		-0.015	0.070		0.911	0.007	0.007	0.008	0.015	0.037	62.0	-0.001	0.000	0.014	0.001
5.15E+05		88.2	3.8	23.2	0.18	0.14	0.01	0.33	15.2	0.04	0.29		0.030	0.120		1.135	0.017	0.017	0.018	0.021	0.073	53.5	0.001	0.000	-0.003	-0.006
4.41E+05		88.2	3.8	23.2	0.18	0.14	0.01	0.33		0.04	0.29		0.376	0.193		1.489	0.017	0.017	0.018	0.021	0.073	53.5	0.002	0.001	0.000	-0.004
4.92E+05		88.2	3.8	23.2	0.18	0.14	0.01	0.33	20.7	0.04	0.29		0.015	0.044		1.855	0.017	0.017	0.018	0.021	0.073	53.5	0.001	0.000	-0.007	-0.009
4.23E+05		104.0	6.5	16.0	0.06	0.16	0.04	0.26		0.29	0.12		-0.216	0.037		-0.827	0.028	0.025	0.019	0.026	0.097	45.9	0.002	0.000	-0.008	-0.009
4.13E+05		104.0	6.5	16.0	0.06	0.16	0.04	0.26		0.29	0.12		-0.301	0.031		-0.682	0.028	0.025	0.019	0.026	0.097	45.9	0.001	-0.001	-0.007	-0.006
5.14E+05		104.0	6.5	16.0	0.06	0.16	0.04	0.26		0.29	0.12		-0.231	0.039		-1.348	0.028	0.025	0.019	0.026	0.097	45.9	0.001	-0.001	-0.006	-0.008
9.18E+05	9.86E+04	77.8	5.59	13.9	0.08	0.02	0.04	0.14	4.2	0.03	0.23	0.018	-0.037	-0.123	-0.032	-0.955	0.007	0.008	0.003	0.005	0.023	35.9	0.001	0.000	0.001	0.000
8.79E+05	8.73E+04	81.2	5.03	16.1	0.08	0.02	0.04	0.14	9.1	0.02	0.13	0.018	-0.027	-0.141	-0.017	0.022	0.007	0.007	0.002	0.005	0.022	32.9	0.000	0.000	0.001	0.000
8.61E+05	8.77E+04	81.3	6.75	12.1	0.08	0.02	0.04	0.14	11.4	0.03	0.18	0.018	-0.032	-0.100	-0.021	-1.904	0.007	0.007	0.002	0.005	0.022	34.5	0.001	0.000	0.001	0.000
7.38E+05	1.27E+05	92.6	5.54	16.7	0.04	0.09	0.39	0.53	8.9	0.03	0.25	0.016	-0.009	-0.135	-0.015	-0.141	0.023	0.020	0.010	0.009	0.062	30.8	0.002	0.000	0.001	-0.001
7.44E+05	1.18E+05	90.5	5.99	15.1	0.04	0.09	0.39	0.53	9.4	0.04	0.17	0.020	-0.021	-0.024	-0.019	0.025	0.023	0.020	0.010	0.010	0.063	31.7	0.002	0.000	0.001	-0.001
7.47E+05	1.22E+05	92.8	5.55	16.7	0.04	0.09	0.39	0.53	2.5	0.04	0.21	0.018	-0.011	-0.122	-0.022	0.403	0.023	0.020	0.010	0.010	0.063	31.3	0.002	0.000	0.001	-0.001
5.77E+05	1.32E+05	138.1	7.06	19.6	0.05	0.13	1.18	1.35	1.8	0.02	0.03	0.006	-0.010	0.074	-0.001	0.239	0.055	0.042	0.024	0.020	0.140	30.8	0.002	-0.001	0.000	0.000
5.16E+05	1.33E+05	135.3	7.75	17.5	0.05	0.13	1.18	1.35	5.1	0.01	0.01		0.001	0.001	0.038	-1.356	0.055	0.042	0.023	0.019	0.139	30.3	0.003	-0.001	0.001	-0.001
5.37E+05	1.29E+05	138.4	7	19.8	0.05	0.13	1.18	1.35	2.7	0.04	0.05	0.014	-0.027	-0.039	-0.004	0.050	0.055	0.042	0.023	0.019	0.140	30.6	0.002	-0.001	0.000	-0.001
5.32E+05	8.33E+04	75.9	5.37	14.1	0.04	0.02	0.02	0.08	6.6	0.02	0.07	0.007	-0.015	-0.012	0.030		0.009	0.010	0.006	0.007	0.031	40.8	0.002	0.000	-0.002	-0.001
5.61E+05	1.38E+05	79.7	4.72	16.9	0.04	0.02	0.02	0.08	1.9	0.03	0.13	0.069	-0.027	-0.102	-0.051	0.112	0.009	0.010	0.005	0.006	0.030	36.2	0.001	0.000	0.005	0.000
5.11E+05	1.53E+05	80.1	4.3	18.6	0.04	0.02	0.02	0.08	1.3	0.14	0.21	0.074	-0.141	-0.165	-0.064	0.084	0.009	0.010	0.004	0.007	0.029	35.7	0.001	0.000	0.007	-0.002
7.02E+05	1.26E+05	101.2	6.4	15.8	0.12	0.15	0.14	0.41		0.08	0.85	0.040	-0.036	-0.143	-0.064	-0.778	0.037	0.032	0.018	0.013	0.101	31.3	-0.002	-0.003	-0.003	-0.005
7.18E+05	1.31E+05	102.5	6.2	16.5	0.12	0.15	0.14	0.41	4.7	0.07	0.84	0.034	-0.031	-0.135	-0.048	-0.145	0.038	0.032	0.021	0.014	0.104	33.0	-0.003	-0.003	-0.006	-0.005
6.50E+05	1.25E+05	102.3	6.36	16.1	0.12	0.15	0.14	0.41	2.6	0.09	0.83	0.021	-0.054	-0.132	-0.049	-0.625	0.037	0.032	0.017	0.013	0.100	31.0	-0.002	-0.003	0.000	-0.006
7.32E+05	1.38E+05	130.3	8.68	15.0	0.15	0.15	0.12	0.41	5.5	0.03	0.11	0.018	-0.007	-0.017	-0.014	-0.508	0.077	0.059	0.031	0.019	0.186	27.2	0.005	-0.001	0.022	-0.001
6.85E+05	1.90E+05	132.5	8.95	14.8	0.15	0.15	0.12	0.41	3.4	0.06	0.14	0.057	-0.045	-0.018	-0.047	-0.305	0.077	0.059	0.031	0.020	0.188	27.4	0.004	-0.002	0.014	-0.003
6.61E+05	2.51E+05	130.8	8.94	14.6	0.15	0.15	0.12	0.41		0.03	0.10	0.043	-0.020	0.034	-0.046	-0.393	0.077	0.059	0.032	0.020	0.188	27.9	0.003	-0.002	-0.002	-0.003
4.17E+05	6.33E+04	78.3	4.36	17.9	0.2	0.04	0.01	0.26	2.1	0.01	0.31	0.091	0.001	0.451	-0.085	0.048	0.008	0.007	0.050	0.011	0.076	80.8	0.001	0.001	-0.020	-0.002
5.97E+05	6.69E+04	76.0	4.78	15.9	0.2	0.04	0.01	0.26		0.01	0.30	0.040	-0.013	0.132	-0.028	-0.199	0.007	0.007	0.036	0.014	0.064	78.6	0.001	0.000	0.000	-0.003
4.42E+05	7.05E+04	77.3	4.28	18.1	0.2	0.04	0.01	0.26	17.5	0.01	0.51	0.026	-0.005	0.374	-0.014	-0.848	0.007	0.007	0.043	0.013	0.070	79.8	0.001	0.000	-0.002	-0.002
7.07E+05	8.21E+04	99.4	5.2	19.1	0.23	0.09	0.03	0.35	2.2	0.08	1.17	0.062	-0.053	-0.105	-0.031	-0.848	0.045	0.028	0.248	0.010	0.331	78.0	0.001	-0.001	-0.009	-0.003
6.34E+05	7.67E+04	95.5	5.58	17.1	0.23	0.09	0.03	0.35	5.3	0.06	0.96	0.038	-0.025	0.078	-0.009	0.194	0.043	0.028	0.205	0.007	0.282	75.2	0.002	-0.001	0.026	-0.002
6.86E+05	8.08E+04	97.5	5.02	19.4	0.23	0.09	0.03	0.35	1.7	0.08	1.00	0.088	-0.026	-0.087	-0.028	-0.856	0.044	0.028	0.227	0.009	0.307	76.7	0.001	-0.001	0.009	-0.004
5.05E+05	5.79E+04	113.3	7.78	14.6	0.2	0.14	0.04	0.38	14.4	0.07	0.65	0.028	0.011	0.337	0.058	-0.847	0.068	0.048	0.048	0.019	0.183	36.5	0.006	-0.001	0.015	-0.002
5.13E+05	7.17E+04	114.6	7	16.4	0.2	0.14	0.04	0.38	16.2	0.05	0.57	0.021	0.030	0.201	0.052	0.330	0.068	0.048	0.039	0.020	0.175	33.7	0.005	-0.001	-0.002	-0.005
4.88E+05	7.71E+04	114.2	7.26	15.7	0.2	0.14	0.04	0.38	9.2	0.08	0.71	0.068	-0.003	0.015	0.116	-0.492	0.068	0.048	0.043	0.019	0.179	35.1	0.004	-0.001	0.008	-0.002

Month	
Station	(triplicates)
Temp	Temperature (°C)
Tot abu t0	Initial total abundance (cells mL^{-1})
LNA abu t0	Initial LNA abundance (cells mL^{-1})
HNA abu t0	Initial HNA abundance (cells mL^{-1})
%HNA	relative contribution of HNA cells
Live abu t0	Initial *Live* abundance (cells mL^{-1})
%Live	relative contribution of *Live* cells
CTC+ abu t0	Initial CTC+ abundance (cells mL^{-1})
%CTC+	relative contribution of CTC+ cells
µ Comm	Specific growth rate Community treatment (d^{-1})
µ Filt	Specific growth rate Filtered treatment (d^{-1})
µ LNA	Specific growth rate LNA cells (d^{-1})
µ HNA	Specific growth rate HNA cells (d^{-1})
µ *Live*	Specific growth rate *Live* cells (d^{-1})
µ CTC+	Specific growth rate CTC+ cells (d^{-1})
K Comm	Carrying capacity Community treatment (cells mL^{-1})
K Filt	Carrying capacity Filtered treatment (cells mL^{-1})
K F/C	Ratio of Community to Filtered treatment carrying capacities
K LNA	Carrying capacity LNA (cells mL^{-1})
K HNA	Carrying capacity HNA (cells mL^{-1})
K *Live*	Carrying capacity *Live* (cells mL^{-1})
K CTC+	Carrying capacity CTC+ (cells mL^{-1})
DOC	DOC concentration (µmol C L^{-1})
DON	DON concentration (µmol N L^{-1})
C:N	DOC:DOC concentration ratio
pChl *a*	picoplankton chlorophyll *a* concentration (µg L^{-1})
nChl *a*	nanoplankton chlorophyll *a* concentration (µg L^{-1})
mChl *a*	microplankton chlorophyll *a* concentration (µg L^{-1})
Chl *a*	total chlorophyll *a* concentration (µg L^{-1})
BGE	bacterial growth efficiency (%)
NO_2^-	nitrite concentration (µmol L^{-1})
NO_3^-	nitrate concentration (µmol L^{-1})
PO_4^{3-}	phosphate concentration (µmol L^{-1})
NO_2^- c.r.	nitrate consumption rate (µmol L^{-1} d^{-1})
NO_3^- c.r.	nitrite consumption rate (µmol L^{-1} d^{-1})
PO_4^{3-} c.r	phosphate consumption rate (µmol L^{-1} d^{-1})
DON c.r	DON consumption rate (µmol L^{-1} d^{-1})
C1	FDOM component C1 concentration (R.U.)
C2	FDOM component C2 concentration (R.U.)
C3	FDOM component C1 concentration (R.U.)
C4	FDOM component C2 concentration (R.U.)
FDOM	Total FDOM components (C1+C2+C3+C4, R.U.)
%protein	Relative contribution of FDOM components C3 and C4 (%)
C1 c.r.	C1 consumption rate (R.U. d^{-1})

C2 c.r.	C2 consumption rate (R.U. d^{-1})
C3 c.r.	C3 consumption rate (R.U. d^{-1})
C4 c.r.	C4 consumption rate (R.U. d^{-1})
